# Resveratrol‐loaded nanomedicines for cancer applications

**DOI:** 10.1002/cnr2.1353

**Published:** 2021-03-02

**Authors:** Manjusha Annaji, Ishwor Poudel, Sai H. S. Boddu, Robert D. Arnold, Amit K. Tiwari, R. Jayachandra Babu

**Affiliations:** ^1^ Department of Drug Discovery and Development Auburn University Auburn Alabama USA; ^2^ Department of Pharmaceutical Sciences, College of Pharmacy and Health Sciences Ajman University Ajman United Arab Emirates; ^3^ Department of Pharmacology and Experimental Therapeutics, College of Pharmacy & Pharmaceutical Sciences University of Toledo Toledo Ohio USA

**Keywords:** cancer, in vitro, in vivo, nanoparticles, polyphenol, resveratrol

## Abstract

**Background:**

Resveratrol (3, 5, 4^′^‐trihydroxystilbene), a natural polyphenol and phytoalexin, has drawn considerable attention in the past decade due to its wide variety of therapeutic activities such as anticancer, anti‐inflammatory, and antioxidant properties. However, its poor water solubility, low chemical stability, and short biological half‐life limit its clinical utility.

**Recent findings:**

Nanoparticles overcome the limitations associated with conventional chemotherapeutic drugs, such as limited availability of drugs to the tumor tissues, high systemic exposures, and consequent toxicity to healthy tissues. This review focuses on the physicochemical properties of resveratrol, the therapeutic potential of resveratrol nano‐formulations, and the anticancer activity of resveratrol encapsulated nanoparticles on various malignancies such as skin, breast, prostate, colon, liver, ovarian, and lung cancers (focusing on both in vitro and in vivo studies).

**Conclusions:**

Nanotechnology approaches have been extensively utilized to achieve higher solubility, improved oral bioavailability, enhanced stability, and controlled release of resveratrol. The resveratrol nanoparticles have markedly enhanced its anticancer activity both in vitro and in vivo, thus considering it as a potential strategy to fight various cancers.

## 1 INTRODUCTION

Traditional medicine, utilizing bioactive natural compounds, has been in use for centuries in various cultures worldwide.^1^ In this regard, naturally occurring dietary phytochemicals have gained significant attention due to their broad range of therapeutic effects in preventing the initiation and progression of a disease. These bioactive natural compounds identified in various foods and beverages are less harmful with excellent therapeutic activity and minimal toxicity. They are inherent within the host system to protect them against viruses, parasites, and various external stresses. Over the past several years, the potential of a wide variety of multi‐targeted phytochemicals such as curcumin, genistein, berberine, resveratrol, quercetin, boswellic acid, epigallocatechin gallate (EGCG), garcinol, piperine, tocotrienol, honokiol, capsaicin, betulinic acid, apigenin, withaferin, and diosgenin has been extensively investigated. Based on the chemical structure, dietary phytochemicals are classified into alkaloids, polyphenols, carotenoids, and nitrogen compounds.^2^ Among the many phytochemicals, polyphenols are reported to contain several bioactive molecules, which are further classified into phenolic acids, flavonoids, stilbenes, coumarins, and lignans. From them, stilbenes, particularly trans‐resveratrol, have gained greater attention due to its wide distribution in the plant kingdom and a broad range of pharmacological activities with multiple signaling pathways and many different targets. The incidence of “French Paradox” demonstrated that consumption of red wine decreases the incidence of cardiovascular diseases despite the intake of a high‐fat diet.^3^ After this highly publicized “French paradox,” resveratrol has gained increased popularity in the scientific community, leading to numerous publications on the investigation of its biological activities. In a study by Soleas et al, the anticarcinogenic properties of four polyphenols were compared. Polyphenols such as catechin, quercetin, gallic acid, and trans‐resveratrol were administered to the mouse twice a week for 18 weeks.^4^ The percentage of tumor inhibition and the number of mice developing one or more tumors were compared among different polyphenols. It was observed that the administration of trans‐resveratrol showed much higher absorption compared to catechin and quercetin. Absorption of trans‐resveratrol is approximately 20‐fold more effective than catechin.^5^ By considering the concentration of polyphenols in their respective dietary sources, it was concluded that trans‐resveratrol is the most effective anticancer polyphenol available in red wine.^4^

Resveratrol (3, 5, 4′‐trihydroxystilbene, a phytoalexin), a natural polyphenol, is found in a wide variety of plants, such as peanuts, blueberries, cranberries, legumes, rhubarb, grapes, eucalyptus, and various grasses. Although resveratrol is naturally occurring, it can only be isolated in a few milligram quantities per kilogram of the plant material, for example, grape skin. Therefore, resveratrol has been chemically synthesized in its purest form for biological use. It has a wide variety of pharmacological activities such as cardioprotection, platelet de‐aggregation, antioxidant, anti‐inflammatory, and vasorelaxant properties.^6^ It also shows antiviral activity against human immunodeficiency virus and the herpes simplex virus,^7^, ^8^ and enhances the antiviral activity of zidovudine, zalcitabine, and didanosine.^9^ One of the main biological activities of resveratrol is that it exhibits anticancer activities against various cancers, which was first reported by Jang et al in the year 1997.^10^

**FIGURE 1:**
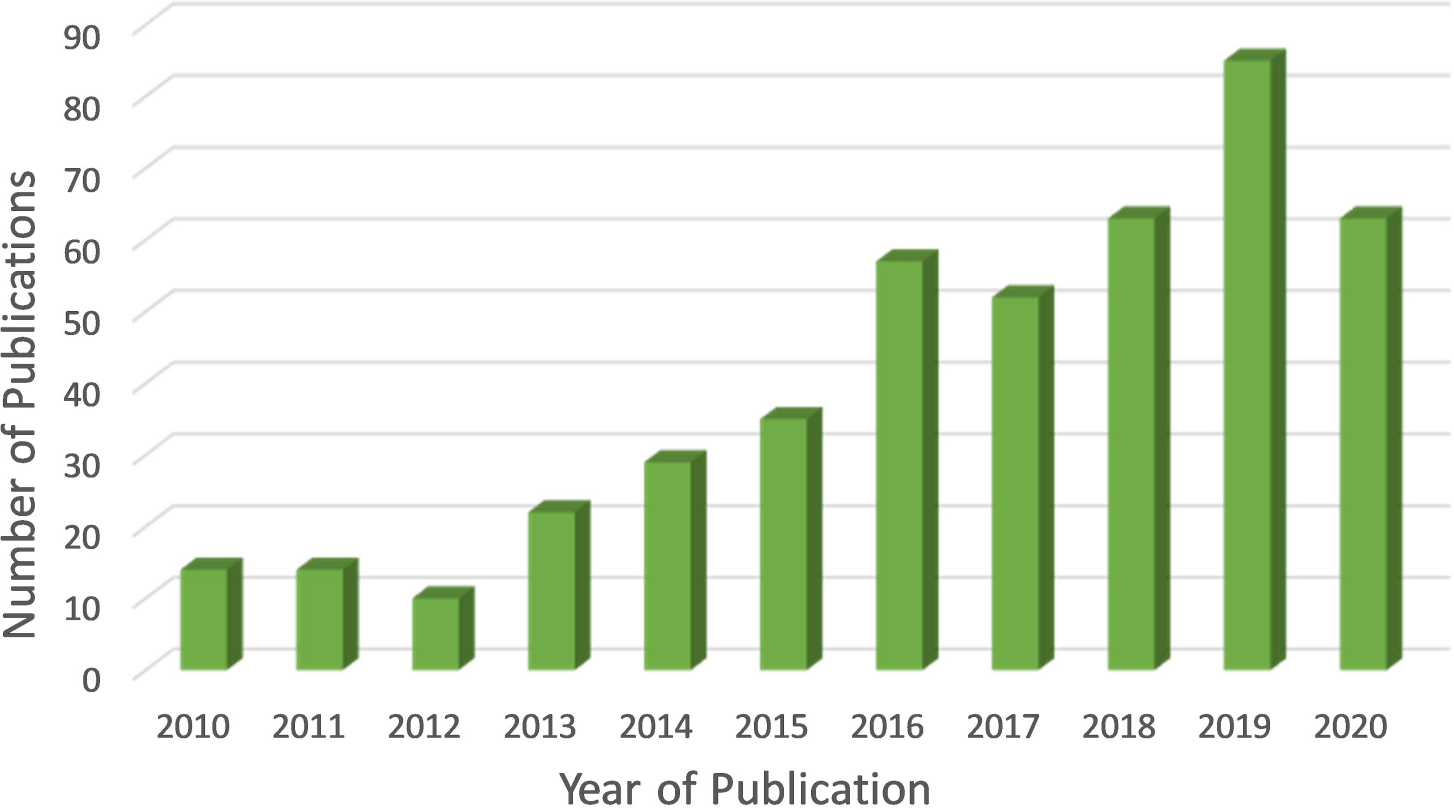
Number of publications in the past decade retrieved using the search terms “Resveratrol”, “nano”, and “cancer” used together from Web of Science (accessed on 10th December 2020)

According to a report from the World Health Organization (WHO), cancer is the second leading cause of death worldwide, accounting for nearly 9.6 million deaths in the year 2018.^11^ Naturally occurring polyphenols have been used both as an adjunct therapy and chemopreventive dietary supplement for decades. In addition, the anticancer activity of resveratrol is reported to be enhanced when used as combination therapy with other chemotherapeutic drugs.^12^, ^13^, ^14^ Several studies have reported the antiproliferative effects of resveratrol in vitro, but the literature is lacking in correlating these results in animal models to enable human application. The low aqueous solubility, chemical instability, and poor absorption across biological membranes limit resveratrol's usage as a chemopreventive or therapeutic agent. Although resveratrol is currently marketed in various traditional dosage forms (tablets, capsules, and powders), there is a lack of sufficient data on its efficacy against cancer prevention and treatment.^15^ To overcome these limitations, nanoparticle‐based formulations have been developed for enhanced absorption and to deliver the optimal concentrations of resveratrol to the tumor target tissue. The novel nano‐formulations for resveratrol delivery include polymeric nanoparticles, liposomes, micelles, metallic nanoparticles, and solid lipid nanoparticles. These systems increase water solubility, stability, and permeation across biological membranes and provide enhanced permeation and retention effect (EPR) in the tumor sites.^16^ The present review attempts to gather comprehensive information on the nanomedicine approach in treating a larger body of cancers. Various challenges in the formulation and delivery of resveratrol and the in vitro and in vivo effects of nanoformulation on each cancer type are presented. Figure 1 shows a rapidly growing trend of resveratrol nanoformulations in cancer for the past decade, showing hundreds of publications in the scientific literature.

## 2 PHYSICOCHEMICAL PROPERTIES AND PHARMACOKINETICS OF RESVERATROL

Resveratrol has a molecular weight of 228.25 g/mol and a melting point of 254°C. It is a creamy white powder, a hydrophobic compound with a log *P*_O/W_ = 3.1,^17^ and low aqueous solubility of 30 μg/mL.^18^ Resveratrol demonstrates a solubility‐limited absorption across biological membranes, and hence it is categorized as a “Class II” compound according to the Biopharmaceutical Classification System. A double bond linking two phenolic rings in the resveratrol structure facilitates the formation of *trans*‐ and *cis*‐isomers, among which the *trans*‐isomer is the most stable form.^19^ The *trans*‐ and *cis*‐isomers exhibit differences in the spectrophotometric UV absorption levels, enabling their identification, and these can be distinguished clearly in nuclear magnetic resonance spectroscopy due to their chemical shifts. Trans‐resveratrol is more biologically active and is converted to the *cis*‐isomeric form upon exposure to UV light.^20^, ^21^ The trans‐resveratrol is stable for months when protected from light in a wide pH range.^21^ The pKa of trans‐resveratrol corresponding to 1, 2, and 3 phenolic groups are 8.99, 9.63, and 10.64, respectively.^22^ Based on the stable nature and biological activity, when the structure of resveratrol is not specified, the compound is generally referred to as trans‐resveratrol.

Despite its chemopreventive properties, resveratrol poses various pharmacokinetic challenges due to its low bioavailability and chemical instability. Following oral administration, resveratrol is well absorbed (~75%) by the intestinal epithelium through passive diffusion. However, it is extensively metabolized in the intestine and liver (glucuronidation and sulfate conjugation) to form metabolites such as trans‐resveratrol‐3‐O‐glucuronide and trans‐resveratrol‐3‐sulfate, respectively.^23^ It can also be found as free resveratrol forming complexes with the low‐density lipoproteins, plasma proteins such as albumin, thus leaving only trace amounts of free resveratrol in the systemic circulation.^24^ It was found that the order of abundance of resveratrol metabolites in the systemic circulation is glucuronides, followed by sulfates, followed by the free resveratrol.^25^ Thus, resveratrol has a very short plasma half‐life of only 8 to 14 minutes,^26^ and reaches peak plasma concentrations at 1 hour (following ingestion) and 6 hours (following enteric recirculation of resveratrol metabolites).^27^ The most significant route of excretion is via urine or feces. However, the excretion of sulfates through urine is higher (84%) than glucuronides and free resveratrol (trace amounts to 17%).^28^ In addition, resveratrol, and its metabolites are also found in feces, where only small quantities of sulfates (<1%) are excreted via feces.^28^ Following intravenous administration of resveratrol, the terminal elimination half‐life ranges between 7.8 and 35 minutes.^29^ In a study by Walle et al (2004), following the intravenous administration of the low dose of ^14^C‐labelled resveratrol, high absorption rate followed by a rapid decline in the peak plasma concentration after 1 hour indicates that the distribution is rapid and only trace amounts of resveratrol enter the enterohepatic circulation. The calculated t_1/2_ after oral and i.v administration is 9.2 and 11.4 hours, respectively,^28^ indicating the superiority of i.v compared to oral administration in overcoming some of the limitations. However, elimination rate and clearance are still faster due to the metabolic instability favoring the conjugation of resveratrol with either glucuronic and/or sulfonic acid.^30^ This rapid metabolization impairs the anticancer efficacy of resveratrol, which could be addressed by developing various nanoformulations with sustained and site‐specific resveratrol delivery.

## 3 NANOTECHNOLOGY FOR DELIVERY OF RESVERATROL

One of the main drawbacks of current cancer therapy is the lack of targeted delivery to the cancer tissue. Due to the high toxicity of conventional chemotherapeutic agents and poor drug delivery, nanomedicines have emerged as a novel tool to improve cancer treatment. Based on the drug's physicochemical properties, nano‐formulations with improved stability, greater circulation half‐lives, enhanced intratumor deposition, and controlled release can be achieved.^31^, ^32^, ^33^ These properties can be optimized to improve antitumor activity and reduce toxicity to nontarget, healthy tissues.^34^, ^35^ Furthermore, imaging probes can be included in the nanoparticles so that the side effects of drugs can be predicted in certain patients by providing data on potential nontarget accumulation sites in healthy tissue.^36^

**FIGURE 2:**
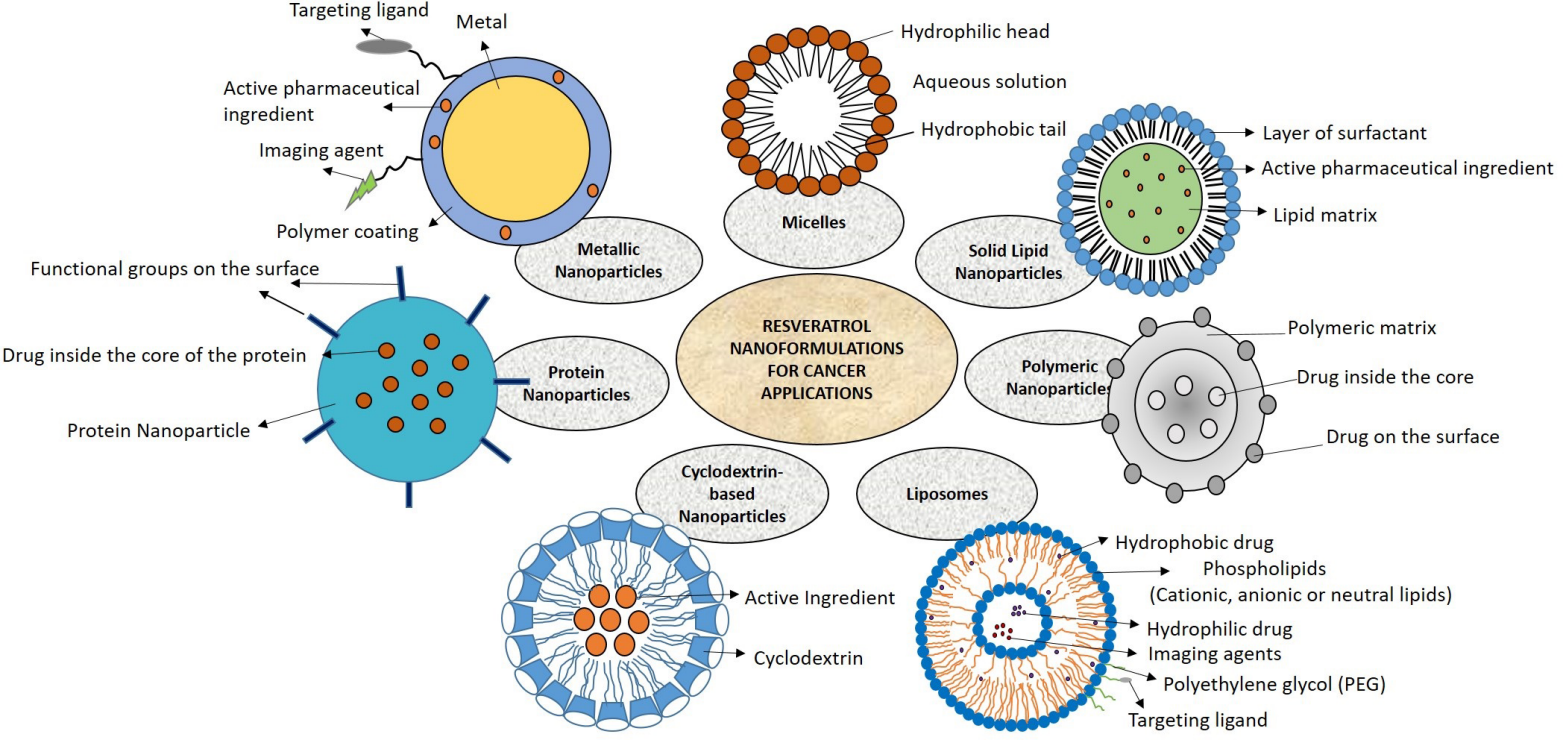
Different types of resveratrol‐loaded nanoparticles for cancer prevention and therapy

Nanomedicines are generally defined as particles that are complex systems consisting of at least two components, one of which is the active ingredient. These nanomedicines typically have particles around 100 to 200 nm nanometers in size and are associated with other pharmaceutical ingredients for stabilizing the formulation or altering the pharmacokinetics (ADME—absorption, distribution, metabolism, and elimination) and improving the drug delivery to the tumor sites.^37^, ^38^ Targeted drug delivery or active targeting describes the specific interaction between the drug carrier and target cells, usually through specific ligand‐receptor interactions,^39^ that generally facilitate the intracellular uptake of nanoparticles. The efficiency of ligand‐receptor binding depends on various factors such as its availability, selective expression of receptor on the target cells, and shedding of the receptor following ligand binding.^36^, ^40^, ^41^ Despite very low bioavailability, considering its therapeutic benefits, resveratrol is available as a dietary supplement in the form of oral dose products such as tablets, capsules, and powders. Nanomedicines have been used to improve bioavailability, reduce metabolism, and improve the delivery of resveratrol.^9^, ^42^, ^43^, ^44^ In addition, they offer advantages such as enhanced tumor targeting, improved solubility, and chemical stability for resveratrol.^45^ A wide range of nanomaterials has been employed in the development of resveratrol cancer therapeutics such as lipids, synthetic polymers, glycan, and proteins. Figure 2 shows various resveratrol nanocarrier systems for cancer treatment. Different biocompatible and biodegradable polymers can be utilized in the preparation of nanoparticles. In general, these nanoparticles are coated with polyethylene glycol (PEG) on their surface, a process called as PEGylation. The PEGylated particles remain in the circulation for extended periods, resist biotransformation reactions, and selectively accumulate in the tumors through the EPR effect.^46^ This higher accumulation in tumor tissues and lower retention in healthy tissues lead to better efficacy with minimal side effects.^47^ More recently, with the help of surface engineering, nanoparticles are conjugated with various targeting ligands such as peptides, antibodies, and aptamers, which allow to reach the targeted tumor directly and release the payload for enhanced efficacy and reduced toxicity.^46^

Liposomes are spherical vesicles containing an aqueous core and phospholipid bilayer and can be differentiated into small unilamellar, giant unilamellar, large unilamellar, and multilamellar based on their size and the number of bilayers.^48^ They can incorporate hydrophilic drugs in the aqueous core and lipophilic drugs in the phospholipid bilayer. Moreover, liposomes can provide protection against photodegradation for various drugs and biochemicals. For example, trans‐resveratrol encapsulated in liposomes remained intact (70% retained) for 16 minutes when exposed to UV light compared to the free drug (10% retained).^49^ Polymeric nanoparticles are another type of drug delivery system where the drug is either conjugated or dispersed within the polymer matrix, protects the drug from degradation, provides a sustained release, and improves bioavailability.^50^ However, another carrier system referred to as solid lipid nanoparticles (SLNs) provides combined benefits of both polymeric nanoparticles and lipid emulsions. They are the spherical vesicles containing lipid core surrounded by hydrophilic surfaces. Generally, hydrophobic drugs such as resveratrol can be easily incorporated into the lipid core.^51^ SLNs have been reported to be superior to liposomes in increasing the chemical stability of resveratrol. It is found to protect against oxidation, hydrolysis, and photodegradation while enhancing the bioavailability of resveratrol.^52^ In addition, surface properties can be altered to modify the uptake. In a study by Teskac et al (2010), cellular uptake, transport, and internalization of resveratrol‐loaded SLN were investigated, which showed that the particles crossed the cell membrane in less than 15 minutes.^52^ Another type of nanocarrier system is cyclodextrins (CD), which are cyclic oligosaccharides containing lipophilic core and hydrophilic surface. They are typically between 1 and 2 nm and form inclusion complexes with the drugs and can enhance the solubility, bioavailability, and stability of the drug.^53^ In a study by Venuti et al (2014), resveratrol sulfobutylether β‐CD complex (1:1) showed increased solubility and cytotoxicity compared to the cyclodextrin without resveratrol, which showed no effect on cell viability.^54^

Based on the targeting principle, nanotherapeutics are classified into passive targeting, active targeting, stimuli‐responsive systems, and theranostics. Both active and passive targeting strategies are used to improve the targeting of anticancer drugs. However, distribution is dependent on the physicochemical properties of a drug and is limited by its penetration into the tumor tissue.^55^, ^56^ Particles that have long‐circulation half‐lives and remain in the systemic circulation for extended periods of time have been able to exploit the EPR effect and accumulate passively.^57^, ^58^ However, optimal activity is dependent on the stability of drugs within the nanoparticle, drug‐carrier release kinetics, tumor vascular extravasation, and uniform intra‐tumor distribution. Active targeting strategies have been employed to improve intracellular uptake and better control targeting to specific cell populations. In contrast, stimuli sensitive nanomedicines can be designed to release the drug upon a trigger, for example, drugs such as doxorubicin, for which delivery is not pH‐sensitive, can be conjugated with a pH‐sensitive nanomedicine in order to increase the cellular uptake and intracellular drug release.^59^ These nanomedicines also decrease tumor resistance to anticancer drugs, thus mediating the stimuli‐responsive drug release and endocytic drug uptake.^60^, ^61^ In the below sections, we will discuss resveratrol delivery systems for various cancer types.

## 4 ANTICANCER ACTIVITY OF RESVERATROL NANOFORMULATIONS

Chemoprevention is defined as a reduction or prevention of cancer risk by ingestion of either synthetic or natural compounds with low toxicity that is able to suppress, delay, or reverse carcinogenesis.^62^ Resveratrol has been found to possess many chemoprevention and chemotherapeutic properties.^63^, ^64^ Resveratrol acts by various cell‐signaling pathways such as cell cycle arrest, suppression of cell proliferation, induction of apoptosis, reduction of inflammation, and inhibition of adhesion, invasion, and metastasis.^65^, ^66^, ^67^ Resveratrol's mechanism of action has been widely studied through in vitro,^68^ and in vivo experiments.^63^, ^69^ It has been well reported that resveratrol's antitumor activity is due to multiple mechanisms, including proapoptotic, antiproliferative, anti‐inflammatory, and antiangiogenesis. For example, in stem‐like cells derived from breast cancer cells, resveratrol induces apoptosis by downregulating fatty acid synthase and enhancing proapoptotic genes such as DAPK2 and BNIP3.^70^ Mechanism of action in in vivo studies is much more complicated, where resveratrol has been shown to affect a number of molecular targets based on formulation, cancer type, stage of the disease, dose, and duration of resveratrol present at the target site of action.^71^

Both reactive oxygen species (ROS) and reactive oxygen metabolites (ROM) are involved in the generation of oxidative stress in vivo.^72^, ^73^ Physiological levels of ROS are essential for transcriptional and posttranscriptional cell signaling.^74^ However, excessive production and accumulation of ROS may induce the modification of cellular proteins and nucleic acids with deleterious effects such as DNA damage, inflammation, and promotion of tumor growth.^75^ Evidence suggests that resveratrol serves as a free radical scavenger because of its ability to promote the activity of various antioxidative enzymes.^76^ It has the potential to inhibit lipid peroxidation (induced by Fenton reaction), decrease the oxidative chain complex, scavenger of ROS, and other free radicals.^75^ Estimation of lipid peroxidases is an indicator of free radical damage to cells. The ortho‐diphenoxyl functionality of resveratrol provides antioxidative activity against ROS‐induced lipid peroxidation.^77^, ^78^ Resveratrol is involved in inhibiting lipid peroxidation at various stages of lipid metabolism, such as initiation, propagation, and termination reactions.^78^, ^79^ At the initiation stage, resveratrol prevents the formation of peroxy radicals, and at the termination stage, it decreases the formation of conjugated alkenes by suppressing glutathione's action peroxidase.

A large number of studies reported that resveratrol affects tumor cells both in vitro and in vivo, at all stages of carcinogenesis (tumor initiation, promotion, and progression). The following sections will focus on the anticancer activity of resveratrol nanoformulations against skin, prostate, breast, lung, colon, liver, and ovarian cancers. Table 1 summarizes different types of resveratrol‐loaded nanocarrier systems for cancer therapy, and Table 2 summarizes the various molecular targets and signaling pathways of resveratrol nanoformulations in different types of cancers.

**TABLE 1:** Co‐delivery of resveratrol nano‐formulations with various chemotherapy drugs

Nanocarrier system	Co‐delivery drug combination	Cancer	Important findings
Pegylated nanoliposomes^80^	Resveratrol and 5‐fluorouracil	Head and neck squamous cell carcinoma	Co‐encapsulation of drugs showed different effects on different genes and enhanced the cytotoxicity in comparison to free drug drugs
Shell crosslinked zein nanocapsules^12^	Exemestane and resveratrol	Breast cancer	Nanocapsules enhanced the cytotoxicity against MCF‐7 and 4 T1 breast cancer cells and reduced the tumor volume by 2.4‐fold compared to the free drug combination
Folic acid conjugated nanoparticles^13^	Resveratrol and docetaxel	Prostate cancer	Nanoparticles downregulated the expression of NF‐kB p65, cox‐2, and antiapoptotic genes and exhibited additional cytotoxic effects with the downregulation of survivin and upregulation of cleaved caspase‐3
Co‐encapsulated liposomes^81^	Resveratrol and paclitaxel	Breast cancer	Composite liposomes showed improved cytotoxicity against drug‐resistant MCF‐7/Adr tumor cells in vitro and enhanced the tumor retention of drugs in vivo.
Alginate nanoparticles^82^	Curcumin and resveratrol	Prostate cancer	Curcumin was found to have good cellular uptake from both the solution as well as nanoparticles. Whereas resveratrol showed poor cellular uptake.
Epidermal growth factor conjugated lipid‐polymer hybrid nanoparticles^14^	Docetaxel and resveratrol	Non‐small cell lung cancer	Nanoparticle co‐delivery system showed significant synergistic effect and tumor growth inhibition with lowest systemic toxicity for both in vitro and in vivo studies.
Solid lipid nanoparticles^83^	Curcumin and resveratrol as a complex with gelucire	Colon cancer	Curcumin‐resveratrol‐gelucire (CRG) complex showed better IC50 value than CRG‐cyclodextrin complex
Phytosomal bilayer enveloped casein micelles^84^	Monascus yellow pigments and resveratrol	Breast cancer	Multireservoir nanocarrier system showed superior cell cytotoxicity, reduction of tumor volume, and inhibition of tumor growth biomarkers
Lactobionic/folate dual‐targeted amphiphilc maltodextrin‐based micelles^85^	Sulfasalazine and resveratrol	Liver cancer	The dual‐targeted micelles showed enhanced cytotoxicity and internalization, reduced liver/body weight ratio, inhibition of angiogenesis, and enhanced apoptosis
Polymeric nanocarriers^86^	Resveratrol and docetaxel	Breast cancer	Polymeric micelles exhibited prolonged release profiles and improved anticancer effect compared to individual drugs in vitro
Polymeric micelles^87^	Resveratrol and curcumin co‐administered with doxorubicin	Ovarian cancer	Coadministration mitigated the doxorubicin induced cardiotoxicity by reduction of apoptosis and ROS and improved the potency of doxorubicin in ovarian cancer cells
Novel peptide‐cationic liposomal nanocarrier^88^	Resveratrol and P53 gene	Cervical cancer and breast cancer	Co‐delivery system showed greater tumor inhibition and apoptosis‐inducing activity than resveratrol liposomes or p53 gene liposomes
Ultradeformable liposomes^89^	Resveratrol and 5‐fluorouracil	Non‐melanoma skin cancer	Co‐encapsulation in ultra‐deformable liposomes showed higher anticancer activity and enhanced accumulation in the deeper skin layers compared to both the free drugs and single entrapped agents
Cyclodextrin nanosponge based hydrogel^90^	Resveratrol and curcumin	Breast cancer	Drug loaded nanosponges showed enhanced in vitro release of curcumin and resveratrol by 10 and 2.5‐fold respectively and higher cytotoxicity compared to free drug
Mesoporous silica nanoparticles^91^	Anti‐miR21 and resveratrol	Colon cancer	The nanoparticles containing hyaluronic acid/resveratrol and antimiR21 showed 3‐fold higher tumor regression effect compared to free resveratrol and 2‐fold higher tumor regression compared to resveratrol‐miR21 nanoparticles
Self‐microemulsifying system^92^	Curcumin and resveratrol	Colon cancer	Co‐formulation showed greater antioxidant activity and lower cytotoxicity than the formulation with individual compounds
Lyotropic liquid crystalline nanoparticles^93^	Resveratrol and pemetrexed	Non‐small cell lung cancer	The nanoparticles showed superior cytotoxicity profile with enhanced cellular uptake and tumor growth inhibition via inhibition of angiogenesis and induction of apoptosis
Polymeric micelles^94^	Co‐delivery of quercetin/resveratrol and resveratrol/curcumin	Ovarian cancer	Micellar formulations of resveratrol and curcumin co‐administered with Adriamycin showed significant tumor reduction and thus capable of mitigating Adriamycin induced cardiotoxicity

**TABLE 2:** Various cellular effects and molecular targets of resveratrol nanoformulations involved in carcinogenesis

Cancer	Molecular targets	Cellular effects	Cell line	Reference
Skin cancer	G1/S arrest. Downregulation of Bcl‐2, Bcl‐xL proteins	Inhibition of NF‐kB signaling pathway	SK‐Mel‐28 and Colo‐38	89
	S‐phase arrest and decrease in G2/M phase	Inhibition of cell division	A375	95
	Avoid metastasis and pulmonary hemorrhage	Increased necrotic area and inflammatory infiltrate of melanoma tumor	B16F10	96
Breast cancer	Downregulation of MMP‐9, COX‐2, NF‐kB protein	Inactivation of PI3K/AKt and ERK1/2 and activation of HO‐1 signaling cascade	MCF‐7	97
	G0/G1 arrest, upregulation of Bax and downregulation of cyclin D1, c‐Myc, and Bcl‐2/Bax ratio	Induction of apoptosis and Wnt signaling pathway	MDA‐MB‐231	98
	Downregulation of BCL‐xl, MMP‐9 and HER‐2. Cell cycle arrest at G2/M phase	Induction of apoptosis and inhibition of HRG‐β1 signaling pathway	T47D and MCF‐7	99
	Suppression of VEGF, CD‐1, aromatase, NF‐kB, and elevation of caspase‐3	Induction of apoptosis, reduction of cell proliferation, and inhibition of PI3K/Akt pathway	MCF‐7	84
Prostate cancer	Reduced expression of Akt protein	Inhibition of microRNA21/Akt signaling pathway	DU‐145, PC3, LNCaP	50
	Downregulation of NF‐kB, p65, COX‐2, BCL‐2, BCL‐XL, survivin, and upregulation of caspase‐3, BAX, BAK	Inhibition of NF‐kB pathway	PC3, C4‐2B, and LNCaP	13
	Cell cycle arrest at G1‐S transition phase and upregulation of caspase‐3	Induction of apoptosis	LNCaP	100
	Increased expression of P53. Reduced expression of MMP‐2 and MMP‐9 and controls angiogenesis	Induction of apoptosis	DU145	82
	Downregulation of Bcl‐2 expression	Induction of apoptosis	PC‐3	101
	Downregulation of p‐Akt, cyclin D1, and m‐TOR proteins	Induction of apoptosis and inhibition of AR/mTOR signaling pathway	PTEN‐CaP8	102
Colon cancer	Decreased expression of intracellular apoptotic protein I (cIAP1)	Inhibition of NF‐kB signaling pathway	HT‐29 and LS147T	103
	Downregulation of cyclin D1	Induction of apoptosis and cell necrosis PI3K/PTEN/Akt pathway	BGC823 and SGC‐7901	91
	Downregulation of NF‐kB and IL‐6	Induction of apoptosis	RAW 264.7 and Caco‐2	104
	Dose‐dependent increase in caspase‐3 and PARP	Induction of apoptosis through activation of P53	CT26	105
Liver cancer	Modulates NO/NOS by upregulating the NO production and NOS activity	Induction of apoptosis and inhibition of PI3K/Akt signaling pathway	SMMC 7721 and L02	106
Lung cancer	Upregulation of Bax, p53, p21, caspase‐3 and downregulation of Bcl‐2 and NF‐kB proteins	Induction of apoptosis and cell cycle arrest in G0/G1 phase	NCI‐H460	107
	Upregulation of caspase‐9 and caspase‐3	Induction of apoptosis in both non‐resistant and resistant cancer cells via mitochondria‐dependent signaling pathway	A549	108
	Increased intracellular ROS generation and DNA damage	Activation of p53 dependent apoptotic cascade	NCI‐H460	109
	Upregulation of caspase‐3 and reduced expression of Ki‐67 and VEGF	Induction of apoptosis and inhibition of angiogenesis	A549	93
Ovarian cancer	Dose‐dependent induction of apoptosis and activation of caspase‐3	Apoptosis inducing factor (AIF) apoptosis pathway	SKOV3	110
	Increased expression of caspase‐3	Induction of apoptosis	ES2‐luc, A2780	94
	Upregulation of Bax, caspase‐9, and downregulation of Bcl‐2	Induction of apoptosis via ROS generation and targeting through mitochondria mediated pathway	PA1	111

Abbreviations: COX‐2, Cyclooxygenase‐2; ERK1/2, extracellular signal regulated kinases; HER‐2, human epidermal growth factor receptor 2; HERG‐β1, heregulin‐beta 1; HO‐1, hemeoxygenase‐1; MAPK, mitogen‐activated protein kinases; MMP‐9, matrix metalloproteinase‐9; mTOR, mammalian target of rapamycin; NF‐kB, nuclear factor kappa; NO, nitric oxide; NOS, nitric oxide synthase; PARP, Poly(ADP‐ribose) polymerase; PI3K/Akt, Phosphatidlyinositol‐3kinase; ROS, reactive oxygen species; VEGF, vascular endothelial growth factor.

### 4.1 Skin cancer

Among all human malignancies, skin cancer is the most common form,^112^ accounting for more than three million cases every year in the United States alone.^113^ The development of skin cancer is related to two main factors, ultraviolet B (UVB) radiation exposure and nuclear factor kappa B (NFkB). Resveratrol, due to its antioxidant properties, can block the damage caused by UVB exposure, thus inhibiting UVB‐induced lipid peroxidase or blocking UV‐mediated activation of NFkB.^10^ Tyrosinase is an essential enzyme for melanin production. Inhibiting this enzyme activity was found to be very effective in controlling melanoma cell growth.^112^ Application of resveratrol, both before and after the UVB exposure drastically reduced the skin damage and decreased skin cancer incidence.^114^ It is able to reduce tyrosinase activity by 30 to 45%.^115^ Resveratrol also inhibits tumor progression by suppressing the growth of various cancer cell types by inhibiting DNA polymerase, deoxy‐ribonucleotide synthesis, and inducing cell cycle arrest.^116^

#### 4.1.1 In vitro studies

In a study by Rigon et al (2016), in vitro biological activity of trans‐resveratrol loaded SLNs was evaluated in various skin disorders. Resveratrol‐loaded SLNs showed a mean particle size less than 200 nm, zeta potential of ~3 mV, and permeated 45% of resveratrol after 24 hours. The nanoparticles also achieved tyrosinase inhibitory activity, greater than or equal to that of the positive control, kojic acid, and proved to be nontoxic in HaCat keratinocytes.^112^ In another study, resveratrol‐loaded nanostructured lipid carriers (NLCs) were prepared with two different lipids, glyceryl behenate (more hydrophobic) and polyoxyethylene 40 (PEG 40) stearate. PEG‐40 stearate based NLCs showed smaller particle size and polydispersity index (PDI) and higher encapsulation efficiency compared to glyceryl behenate. In addition, both the formulations showed very less release due to the crystalline nature of the lipid matrix. It was also reported that resveratrol cytotoxicity against the L‐929 fibroblast cell line did not increase when loaded into NLCs. Interestingly, resveratrol formulation prepared with PEG‐40 stearate showed 1.31 and 1.83 times higher inhibition of tyrosinase than resveratrol solution and formulation containing glyceryl behenate, respectively.^117^ In a similar study, glyceryl dibehenate based SLNs were formulated, which showed a mean particle diameter of 180 nm, PDI of 0.3, and zeta potential of ‐38 mV suggesting good physical stability of nanoparticles. Moreover, these nanoparticles delivered resveratrol in a biphasic pattern with about 40% rapid release (drug inside the shell) followed by a sustained release (drug inside the lipid matrix). In addition, these nanoparticles expressed the cytostatic effects with a large drop of the G2/M phase and the cell cycle arrest at s‐phase. These drug‐encapsulated SLNs showed higher solubility, stability, and greater cytotoxic effects compared to the solution indicating their effectiveness as a nanocarrier system.^52^ In another study, different nanocarrier systems such as liposomes, polymeric lipid‐core nanocapsules, and nanospheres were developed for E‐resveratrol. The degree of photostability of these nanocarriers was compared with the ethanolic solution of E‐resveratrol. The nanostructures were capable of enhancing the E‐resveratrol chemical photostability. The lipid‐core nanocapsules (LCN) and NLC had the same isomerization rate. However, liposomes are the particles that have highly protected E‐resveratrol from photoisomerization but have poor physical stability, resulting in a bimodal size distribution profile. LCNs and NLCs presented similar penetration profiles under dark conditions. When exposed to ultraviolet A (UVA) radiation, nanocarriers led to higher concentrations of E‐resveratrol in the total epidermis, confirming the skin targeting effect of these nanocarriers.^118^ Based on the analysis, it is important to consider the nanocarriers, which can prevent the degradation of photosensitive drugs. Liposomes were found to provide the highest photostability (only 29.3% isomerization) among all the nanocarriers tested. However, resveratrol‐loaded liposomes were found to be unstable after 8 hours of UV exposure. Therefore, it was essential to store resveratrol‐loaded liposome formulations away from light. Similarly, Freidrich et al (2015) formulated sorbitan monostearate‐based lipid‐core nanocapsules containing both resveratrol and curcumin. This formulation did not show any cell toxicity to dermal fibroblasts. The uptake of nanocapsules within 24 hours into the skin suggests this delivery system would be appropriate for topical delivery. In vitro drug release profile showed a faster release of resveratrol than curcumin. Increased penetration into deeper skin layers was observed with co‐delivery compared to the individual drug encapsulation. It was speculated that the lipophilicity of curcumin facilitated the enhanced delivery of resveratrol across the skin.^119^ Similarly, Cosco et al (2015) studied the ultra‐deformable (elastic) liposomes loaded with resveratrol and 5‐fluorouracil (5‐FU) for the treatment of nonmelanoma skin cancer. The epidermal levels of drugs were significantly higher for the drugs encapsulated within liposome as compared to the controls. These co‐drug loaded liposomes showed improved antitumor activity for both drugs based on cytotoxicity, cell‐cycle arrest, and apoptosis assays. This is due to the accumulation of liposomes in deeper skin layers, generating a cutaneous depot from which both the drugs are gradually released. Moreover, resveratrol increased the antiproliferative potential of 5‐FU in Colo‐38 skin cells. Therefore, co‐encapsulation of 5‐FU and resveratrol in ultra‐deformable liposomes could be beneficial for the treatment of squamous cell carcinoma, namely actinic keratosis and Bowen's disease. However, a major drawback is the limited drug loading capacity of co‐encapsulated liposomes. The liposomes can only accommodate a limited amount of drug in the phospholipid bilayer without affecting its structural integrity. Moreover, liposomes have a very short half‐life and require cold temperatures for storage. Therefore, it is important to consider these limitations before utilizing them as drug delivery carriers.^89^ In another study, resveratrol‐coated hollow gold nanoparticles were prepared for improved photothermal performance and cytotoxicity against melanoma cancer. The nanoparticles could block cell cycle to inhibit cell division and lead to cell apoptosis after 808‐nm laser irradiation in A375 melanoma cells. The nanoparticles were surfactant‐free and hence avoid separation procedures for the surfactant, and surface modification processes that are necessary for most theranostics materials.^95^

#### 4.1.2 In vivo studies

The potential of resveratrol in the chemoprevention and treatment of melanoma and other skin cancers was evaluated by some in vivo studies. Jang et al (1997) first reported the chemopreventive role of resveratrol in skin cancer in mice treated with a carcinogen.^10^ In SKH‐1 hairless mice, topically applied resveratrol showed greater inhibition of UVB mediated skin inflammation, induction of cyclooxygenase and ornithine decarboxylase, and generation of hydrogen peroxide in the skin.^120^ Similarly, resveratrol's anticancer activity against multiple UVB exposures and the involvement of survivin was studied in SKH‐1 hairless mouse skin. It was found that topical pretreatment of resveratrol resulted in inhibition of UVB exposure mediated cell proliferation and phosphorylation of survivin.^121^ In a study by Carletto et al (2016), resveratrol‐loaded poly (caprolactone) nanocapsules were formulated for improved antitumor activity in melanoma cancer. All the nanoformulations showed particle size lower than 150 nm, PDI < 0.2, negative zeta potential, and high encapsulation efficiency due to a greater affinity of resveratrol to the oil core. Nanoencapsulation also leads to drug amorphization and improved resveratrol solubility. The nanoencapsulated resveratrol significantly reduced cell viability of B16F10 melanoma cells vs free resveratrol. In a mouse model bearing B16F10 melanoma tumors, the nanoformulation showed decreased tumor volume, increased necrotic area, and inflammatory infiltrate of melanoma and thus prevented metastasis and pulmonary hemorrhage compared to the free resveratrol. Due to the incorporation of hydrophobic polymer such as PCL, the rate of biodegradation was slow compared to other protein‐based polymers. Moreover, the nanocapsules are not surface‐functionalized and rather taken up by the cells by either clathrin‐mediated endocytosis or macro‐pinocytosis.^96^

### 4.2 Breast cancer

Breast cancer represents the second leading cause of cancer death in females after lung cancer.^122^ Both metastasis and invasion are two main properties of breast cancer cells, involving several biological processes such as adhesion, proliferation, and degradation of the basement membrane.^123^, ^124^, ^125^ Resveratrol plays a vital role in the prevention and tumor suppression at all stages (initiation, promotion, and progression) of breast carcinogenesis.^126^ Both its antioxidant and anti‐inflammatory properties contribute significantly to inducing apoptosis and cell cycle arrest in the development of breast cancer.^126^ Resveratrol also has estrogenic activity, which can function as an estrogen receptor (ER) α agonist or antagonistic ligand at very low concentrations.^127^, ^128^ It inhibits the growth, proliferation, invasion, and metastasis by downregulating molecular targets such as matrix metalloproteinase 9 (MMP‐9), cyclooxygenase‐2 (Cox‐2), apoptotic protein1 (AP‐1), and NFkB in various breast cancer cell lines.^129^, ^130^ It has also been shown to induce tumor suppression and increase apoptotic index in ERα − ERβ + MDA‐MB‐231 tumors.^131^

#### 4.2.1 In vitro studies

Vergaro et al (2012) studied the effect of resveratrol‐loaded halloysite clay nanotubes on MCF‐7 human breast cancer cell lines. The nanotubes were saturated with resveratrol to obtain a delivery system with high encapsulation efficiency. It was found that resveratrol induced downregulation of protein cyclin D1 and decreased phosphorylation of two kinases such as protein kinase B and glycogen synthase 3b, which are potentially involved in the regulation of cyclin D1. Resveratrol‐loaded clay nanotubes controlled the release up to 48 hours by using a layer‐by‐layer polyelectrolyte multilayer coating, which strongly increased cell cytotoxicity leading to apoptosis.^132^ Park et al (2016) demonstrated the effect of gold‐conjugated resveratrol nanoparticles on breast cancer metastasis. These nanoparticles showed better anti‐invasive activity as compared to free resveratrol. The inhibitory activity of these nanoparticles on MMP‐9, COX‐2, NFkB, and AP1 was stronger than that of the free resveratrol. The anti‐invasive effect of resveratrol‐loaded gold nanoparticles in response to TPA stimulation is mediated by the downregulation of MMP‐9, COX‐2, NFkB, AP1, ERK, and activation of signaling cascade.^97^ In another study, Wang et al (2017) studied the effect of resveratrol‐loaded SLNs on the MDA‐MB‐231 breast cancer cell line. The nanoparticles were prepared with stearic acid using emulsification and low‐temperature solidification method, which had a desirable size (below 200 nm) and zeta‐potential (−25 mV) with remarkable stability for resveratrol delivery to the breast cancer cells. These nanoparticles significantly increased Bax, decreased the levels of Bcl‐2, cyclin D1, and c‐Myc. The nanoparticles showed superior results to free resveratrol in inducing cancer cell apoptosis and death.^98^ Recently, a co‐delivery system of resveratrol and Herceptin was reported to improve the cytotoxic profile of Herceptin on the T47D (HER‐2 receptor‐positive breast cancer cell line) and MCF‐7 (HER‐2 receptor‐negative breast cancer cell line). It was found that combining Herceptin with resveratrol significantly reduced the expression of the HER‐2 receptor. Moreover, cell cycle was arrested at the G_2_/M phase for both the cell lines.^99^ In another study, cyclodextrin nanosponge based hydrogel loaded with curcumin and resveratrol was developed. The nanosponges enhanced the release of curcumin and resveratrol by 10‐ and 2.5‐fold, respectively, compared to free drugs. Moreover, the drug‐loaded nanosponge showed a synergistic cytotoxic effect with a combination index value of 0.29 in MCF‐7 cells. The nanosponges in a carbopol based hydrogel formulation showed a significant enhancement in photostability and permeation for both curcumin and resveratrol compared to the hydrogel without cyclodextrin based nanosponges. Despite the advantages of cyclodextrin nanosponges in enhancing the aqueous solubility and photostability, their application is limited in cancer therapy due to its potential nephrotoxicity, lack of targeting ability, potential to alter the pharmacokinetics of drug when rapid dissociation does not occur. Therefore, it is important to overcome these limitations in order to potentiate its chemotherapeutic effects.^90^ In another study, a peptide‐cationic lipid (CD014) based liposome formulation for co‐delivering resveratrol and p53 gene was prepared. The antitumor effects of the co‐delivery formulation against Hela and MCF‐7 cells were investigated. The highest transfection efficiency was reported when the lipid/pDNA (N/P) weight ratio was 3/1, and Hela cells had a higher transfection efficiency than MCF‐7 cells. Cytotoxicity assay showed that the co‐delivery system of resveratrol and p53 had more significant inhibition on Hela cells and MCF‐7 cells than the blank liposomes and resveratrol liposomes, whereas MCF‐7 cells exhibited lower cell viability than Hela cells. Thus, the co‐delivery via liposome might be a potential chemotherapeutic agent for the synergistic treatment in breast cancers. This study demonstrates the advantages of utilizing cationic liposomes for improving cellular uptake compared to zwitterionic liposomes. The possible mechanism might be due to enhanced interaction of the delocalized conjugated pi electrons of the polyphenol with the cationic liposomes, causing an enhanced fusion of nanoparticles to the membrane.^88^

#### 4.2.2 In vivo studies

In a study by Meng et al (2016), pegylated liposomes were constructed by co‐encapsulating resveratrol and paclitaxel using phosphatidylcholine and DSPE‐mPEG2000 lipids.^81^ Both drug molecules resided within the aqueous core of the liposome structure. The composite liposomes showed an average diameter of 50 nm, encapsulation efficiency greater than 50%, and generated potent cytotoxicity against MCF‐7/Adr tumor cells. MCF‐7 is a drug‐sensitive cell line, whereas MCF‐7/Adr is a drug‐resistant cell line. Administration of formulation to mice showed 10‐fold higher blood concentrations of paclitaxel and resveratrol, with long circulation times compared to free drug solutions. The formulation also enhanced tumor retention and superior multidrug resistance reversal without significant side effects in vivo. For instance, the liposome reversed the paclitaxel resistance in drug‐resistant cells and improved the efficacy of both drugs against drug‐sensitive and drug‐resistant tumors in vivo. Analysis of the results revealed the importance of surface modification of liposomes with DSPE‐mPEG2000 to obtain smaller particle size, enhance the cellular uptake, and protect the vesicles from metabolic clearance in vivo. Thus, it is essential to incorporate higher content of DSPE‐PEG2000 and repeated freeze‐thaw cycles to obtain liposomes with smaller particle size^81^ Similarly, Elzoghby et al (2017) constructed shell cross‐linked zein nanocapsules for oral co‐delivery of exemestane and resveratrol. This co‐delivery system showed superior cytotoxicity compared to the free drug combination in both MCF‐7 and 4 T1 breast cancer cells. In vivo study demonstrated a marked reduction in tumor volume by 2.4‐fold compared to the free drug combination.^12^ In another study by Poonia et al (2019), resveratrol‐loaded nanostructured lipid carriers (based on stearic acid—oleic acid) as a parenteral formulation for breast cancer treatment was developed.^133^ The nanocarrier was further modified by conjugating to folic acid moiety as a targeting agent. The optimal formulation showed a mean diameter of 88 nm with high encapsulation efficiency (88%). The folate‐targeted nanocarrier revealed high cytotoxic effects compared to unmodified nanocarrier on MCF‐7 cells with high levels of over‐expressed folate receptors, suggesting the enormous potential of targeted nanocarriers in enhancing the therapeutic concentration of resveratrol to breast cancer cells. Intravenous delivery of the nanocarrier formulation in rats demonstrated a 9‐fold increase in the bioavailability for the folate targeted nanocarrier in comparison to free resveratrol.^133^ In a similar study, folic acid functionalized pluronic 127/D‐α‐tocopheryl PEG 1000 succinate mixed micelles loaded with resveratrol were prepared for breast cancer treatment.^134^ The novel mixed micelle system was prepared using the thin‐film hydration method in order to address low solubility, rapid metabolism, and enhance its accumulation at the tumor site. The micelles showed an average diameter of 20 nm, encapsulation efficiency of 99.67%, and showed a sustained‐release behavior as compared with the propylene glycol solution. The micelle formulation also exhibited enhanced cell uptake via folate receptor‐mediated endocytosis. The folic acid conjugated micelles showed 4‐fold higher plasma levels of resveratrol upon i.v administration in rats, compared to solution. Moreover, there was a lower accumulation in the heart and kidney, suggesting lower exposure to other vital organs.^134^ In another study by El‐Far et al (2018), monascin and ankaflavin, the major components of fungal‐derived monascus yellow pigments, were incorporated along with resveratrol in the core of folate‐conjugated casein micelles for active targeted system.^84^ In contrast, as a passive targeting system, PEGylated resveratrol‐phospholipid complex bilayer enveloping casein micelles were developed. The co‐loaded micelles showed higher cytotoxicity than free drugs in MCF‐7 breast cancer cells. Both nanosystems showed excellent antitumor efficacy, with PEGylated micelles showing comparable tumor suppression to folate‐conjugated micelles in tumor‐bearing mice. Therefore, the co‐delivery of monascus yellow pigments and resveratrol‐loaded micelles were found to be effective for breast cancer treatment. Despite its tumor‐targeting abilities, the limitations of polymeric micelles such as lack of stability in the blood and difficulty in scaling up the current synthetic technique need to be carefully evaluated.^84^

### 4.3 Prostate cancer

Prostate adenocarcinoma is the second most frequent cancer among men in the United States.^135^ Some of the significant risks for human prostate cancer development include age‐related factors and genetic mutations such as loss of phosphatase and tensin homolog (PTEN), leading to increased cancer cell proliferation.^136^ Even though resveratrol intake was not associated with the reduction of prostate serum antigen levels, it was found to be effective in lowering the levels of androgen precursors such as androstenolone, as evidenced by the downregulated expression of androgen receptor and kallikrein, an orthologue of human prostate‐specific antigen.^137^ In another recent study, resveratrol inhibited Akt/MicroRNA‐21 pathway, thus reducing the cancer growth and metastasis. The mechanism involves the downregulation of various prostate‐tumor associated microRNAs, including miR‐21 and upregulation of tumor suppressors (PDCD4 and maspin) in DU145 and LNCaP prostate cancer cells.^138^ The anticancer effect of resveratrol nanoformulations in prostate cancer has been well reported in many in vitro and in vivo studies.

#### 4.3.1 In vitro studies

In a study by Sanna et al (2013), resveratrol‐loaded nanoparticles containing poly (epsilon‐caprolactone) (PCL), and poly (D, L‐lactic‐co‐glycolic acid) (PLGA)‐poly(ethylene glycol) conjugate was prepared by nanoprecipitation method.^50^ The nanoparticles were prepared to protect against degradation, enhance the bioavailability, improve the intracellular penetration, and prolong the release of resveratrol. The nanoparticles (mean diameter of 150 nm) showed high encapsulation efficiency (up to 98%) and controlled the release (~50% released in 7 hours) both at pH 6.5 and 7.4, simulating the acidic tumoral microenvironment and physiological conditions, respectively. The nanoparticles significantly improved the antiproliferative efficacy compared to free resveratrol in DU‐145, PC3, and LNCaP cell lines. Moreover, the nanoformulation showed significantly higher cytotoxicity compared to the free drug in all three prostate cancer cell lines.^50^ In a similar study, PLGA nanoparticles encapsulating resveratrol were designed, and their cytotoxic effects were evaluated. The nanoformulation significantly decreased the cell viability, induced apoptosis by mediating cell cycle arrest of the G1‐S transition phase, DNA nicking, loss of mitochondrial membrane potential, and ROS generation in LNCaP cells. Moreover, the nanoparticles showed significantly higher cytotoxicity compared to free resveratrol at all tested concentrations. Adverse cytotoxic effects were not observed in murine macrophages, even at 200 μM. These findings support the further investigation of resveratrol‐loaded nanoparticles for the chemoprevention/therapy of prostate cancer.^100^ Similarly, Saralkar et al (2017) formulated calcium alginate nanoparticles loaded with curcumin and resveratrol and tested the in vitro efficacy against DU‐145 prostate cancer cells. Nanosuspension and freeze‐dried nanoparticles had a particle size around 12 and 60 nm, respectively, with encapsulation efficiency around 49% (for curcumin) and 71% (for resveratrol). Resveratrol showed a faster and higher release (87.6%) than curcumin (16.3%) in 24 hours. The co‐delivery nanoparticles exhibited a more significant cytotoxic effect on DU‐145 prostate cancer cells than the drug solution, and at higher concentrations, the drug solution showed greater toxicity than nanoparticles.^82^ A similar study also demonstrated the synergistic chemotherapeutic effects of resveratrol and docetaxel. A receptor‐based targeted delivery approach by using folic acid conjugated novel planetary ball milled nanoparticle to treat advanced metastatic prostate cancer was developed. The co‐delivery downregulated the genes (ABCB1, ABCC2, and ABCG2) at both mRNA and protein levels and also downregulated the expression of antiapoptotic markers (Bcl‐2, Bcl‐xl), whereas apoptotic markers (Bax, Bak) were upregulated, thereby reducing the growth of prostate cancer cells.^13^ In another study, resveratrol‐loaded SLNs based on stearic acid and tristearin were formulated using the solvent diffusion evaporation method for enhanced delivery of resveratrol. Nanoparticles rapidly moved through the cell membrane, distributed throughout the cytosol, and moved successively among different cellular levels with greater cytotoxicity compared to resveratrol solution in PC3 cells. This intracellular delivery decreased cell proliferation, thereby inducing selective apoptosis in prostate cancer cells. Drug‐loaded SLNs were prepared using a high‐shear homogenization technique, which greatly eliminates the need for organic solvents, thereby avoiding toxicity compared to other techniques such as solvent evaporation or film ultrasound dispersion. Therefore, the method of preparation of nanoparticles is a critical parameter in determining the properties and their behavior both in vitro and in vivo.^101^

#### 4.3.2 In vivo studies

Narayanan et al (2009) developed liposomes encapsulating resveratrol and curcumin. The curcumin liposomes and resveratrol liposomes were prepared separately using 1,2‐dimyristoyl‐rac‐glycero‐3‐phosphocholine, then combined each in a 2.5 mg/kg (1:1) ratio and freeze‐dried. In vitro studies showed that these liposomes effectively inhibited cell growth and induced apoptosis in PTEN‐CaP8 cancer cells. Moreover, the coadministration of resveratrol and curcumin significantly decreased prostatic adenocarcinoma in B6C3F1/J mice. Molecular targets that are activated due to loss of PTEN, including p‐Akt, cyclin D1, and androgen receptor, were downregulated by co‐encapsulation, suggesting that these liposomes can target multiple mechanisms. One such mechanism is due to enhanced binding of coadministered liposomes to the albumin, enhancing its transportation into the bloodstream and thus improving its therapeutic efficacy.^102^

### 4.4 Colon cancer

According to WHO, colon cancer is the fifth most common cause of cancer deaths worldwide.^139^ About 95% of the colorectal cancer cases are caused by common dietary and environmental factors.^140^ Some of the significant factors that influence colorectal cancer are old age, smoking, high alcohol consumption, diabetes mellitus, obesity, and low fiber intake.^141^ Due to poor bioavailability and substantial accumulation of resveratrol in the colon, it is considered as the most convenient target for application.^142^ Arunachalam and coworkers reported that the NFkB pathway is the main contributor to colon cancer. Resveratrol reverses the activation of NFkB, which is responsible for inducing inflammatory cytokines.^143^ Hope et al (2008) reported that resveratrol at low concentrations significantly inhibited Wnt signaling in colon‐derived cells. This inhibitory effect was due to the regulation of intracellular β‐catenin localization.^144^ In a similar study carried by Sakoguchi et al (2007), resveratrol is shown to induce apoptosis and inhibit proliferation by reducing both Wnt/β‐catenin signaling and expression of survivin.^145^

#### 4.4.1 In vitro studies

Resveratrol‐loaded colloidal mesoporous silica nanoparticles were prepared for its enhanced cytotoxicity in colon cancer cells. The nano‐formulation showed enhanced solubility of resveratrol by 2‐fold and sustained the release compared to pure resveratrol. The cytotoxicity of the nanoformulation against HT‐29 and LS147T colon cancer cell lines was significantly higher than that of unformulated resveratrol.^103^ In another study, resveratrol and anti‐miR21 loaded mesoporous silica nanoparticles conjugated with hyaluronic acid were developed for the treatment of gastric carcinoma. The surface conjugation of hyaluronic acid acted as a targeting agent to the overexpressed CD44 receptor on the cancer cells. This system showed higher cytotoxicity and cellular uptake compared to the naive formulations. The targeted nanoformulation showed a synergistic effect due to the co‐delivery of anti‐miR21 and resveratrol in gastric cancer cells. Importantly, the targeted nanoformulation showed a 3‐fold and 2‐fold higher tumor regression effect compared to that of free resveratrol and non‐targeted nanoformulation, respectively. Thus, a co‐delivery system of anti‐miR21 and resveratrol in a targeted nanoformulation could serve as a promising system for the treatment of gastric carcinoma. The possible reason for its enhanced anticancer efficacy was due to surface modification of mesoporous silica nanoparticles with polyethyleneimine (PEI) to incorporate anti‐miR21, which increases the stability and enhances the cancer‐targeting ability. Moreover, surface conjugation with hyaluronic acid increased its specific binding to the tumor receptors, thereby improving its cellular internalization.^91^ In another study by Kamal et al (2018), technetium‐99m labeled resveratrol‐loaded gold nanoparticles (stabilized by gum arabic) were characterized and evaluated for their targeting efficacy in HT29 colon cancer cells and in a rat cancer model. The cellular uptake of the resveratrol gold nanoparticle system was significantly higher than gold nanoparticles or resveratrol alone. Following i.v administration of the resveratrol gold nanoparticle system to colon tumor‐bearing rats showed better in vivo targeting compared to 99m technetium labeled resveratrol.^146^ Similarly, in a study by Juere et al (2017), resveratrol‐loaded mesoporous silica nanospheres were formulated. The saturated solubility of nanosphere‐embedded resveratrol was dependent not only on the pore size but also on the particle size of the nanospheres. The permeability of nanosphere‐loaded resveratrol across human colon carcinoma cell monolayer (Caco‐2) was enhanced compared to a resveratrol suspension. The resveratrol encapsulation also provided higher anti‐inflammatory activity compared to both resveratrol suspension and solution.^104^ In another study by Soo et al (2016), resveratrol is co‐encapsulated with cyclodextrin‐resveratrol inclusion complex in the lipophilic and hydrophilic compartments of liposomes, utilizing a novel dual carrier approach. The co‐encapsulated liposome formulation showed a particle size of 131 nm, PDI of 0.089, and zeta potential of −2.64 mV. Both the free resveratrol and conventional liposomal formulations showed a drug release profile of ~60%. However, the co‐encapsulated liposome formulation showed a 100% drug release in 24 hours. The in vitro cytotoxicity (potency) of liposomes was also significantly enhanced compared to free resveratrol in HT‐29 colon cancer cell lines. Due to limitations of the use of cyclodextrins or liposomes as individual drug delivery vehicles, both cyclodextrins and liposomes were combined into a single delivery system. Despite showing improved delivery and cytotoxicity of resveratrol, further research is required to determine cyclodextrin's effect on the structural integrity of vesicles, maintain the physical stability of liposomes, and prevent metabolic clearance in vivo.^147^

#### 4.4.2 In vivo studies

Resveratrol‐loaded PEG‐polylactic acid‐based polymeric nanoparticles were designed to suppress the glucose metabolism and tumor growth both in vitro and in vivo.^105^ These nanoparticles showed an increased apoptotic cell death and ^18^F‐fluorodeoxyglucose (^18^F‐FDG) uptake and reduced ROS compared to control in CT26 colon cancer cells. Whereas in CT26 tumor‐bearing mice, ^18^F FDG uptake was reduced with retardation of tumor growth and improved survival rate compared to empty nanoparticle‐injected control.^105^ In another study, a novel self‐microemulsifying formulation (SMEDDS) (based on Capryol 90, Cremophor EL) containing curcumin together with resveratrol was developed to address its poor aqueous solubility, improve their absorption, and delivery across colon cancer cells. This co‐delivery system showed higher antioxidant and cytotoxic activity than the nanoemulsion with either curcumin or resveratrol alone, demonstrating synergistic cytotoxic action due to the co‐delivery formulation. Following oral administration of nanoemulsion to rabbits, the total plasma concentrations of curcumin and resveratrol increased by 10‐ and 6‐fold, respectively, compared to the unformulated drug combination. The nanoformulation achieved increased solubility, protection from degradation, and improved the absorption of resveratrol and curcumin. The possible mechanism of action is due to the presence of the drug in the dissolved form in the SMEDDS, and due to its smaller particle size, the surface interfacial tension is increased, thereby enhancing the rate and extent of oral absorption.^92^

### 4.5 Liver cancer

Liver cancer is the fourth leading cause of all cancer‐related deaths worldwide, which is reported to have relatively high mortality and morbidity in men.^148^ Treatment is often challenging due to the high systemic toxicity of chemotherapeutic drugs, leading to discontinuation of the treatment. Therefore, nano‐formulations and targeted delivery of anticancer agents are highly beneficial to enhance the efficacy, increase the uptake and internalization of drugs into tumors, and reduce the toxicity in healthy tissues.

#### 4.5.1 In vitro studies

Resveratrol‐loaded chitosan nanoparticles were surface‐modified with either biotin or both biotin and avidin.^149^ The nanoparticles containing both avidin and biotin demonstrated a size range <200 nm and superior cytotoxicity in HepG2 cells compared to biotin alone.^149^ These nanoparticles enhanced the target specificity of resveratrol‐loaded chitosan nanoparticles in hepatocarcinoma. In addition, it was observed that the drug‐loaded nanoparticles surface modified with both avidin and biotin had a higher liver targeting index (2.70) and more potent cytotoxicity against HepG2 cells than nanoparticles surface modified with biotin alone.^149^ Similarly, resveratrol‐loaded ionically crosslinked chitosan nanoparticles were prepared to improve the stability, solubility, and hepatic tumor targeting of resveratrol. The nanoparticles were able to improve the long‐term stability and drug release in simulated tumor pH 6.5 than at physiological pH 7.4. Moreover, the antioxidant activity of resveratrol was maintained even after UV light irradiation. The nanoparticles were efficiently taken up by hepatocellular carcinoma (SMMC 7721) cells, showed similar antiproliferative activity in SMMC 7721 cells, and lower cytotoxicity in normal hepatocyte cells (L02) compared to free resveratrol. All the abovementioned advantages might be due to the smaller size of chitosan nanoparticles (~200 nm), accumulating the drug at the cancer site by EPR effect.^106^ In another study, resveratrol nanosuspension composed of poloxamer 188 was prepared using a high‐pressure homogenizer, and in vitro anti‐hepatocarcinoma effects relative to free resveratrol was evaluated. The particle size of the nanosuspension was ~159 nm, and the zeta potential was −2.1 mV. The nanosuspension inhibited the proliferation of HepG2 cells (2.5‐fold lower IC50 values) than the bulk resveratrol.^150^ Therefore, resveratrol‐loaded nanoformulations were found to be very promising for the treatment of hepatocarcinoma.

#### 4.5.2 In vivo studies

In a study by Zhang et al (2019) nano‐gold loaded resveratrol was synthesized and evaluated for its antitumor activity in liver cancer cells and tumor xenografts. The gold‐resveratrol nanoparticles effectively inhibited cell proliferation and promoted apoptosis in HepG2 cells compared to free resveratrol by the downregulation of pro‐caspase‐9, pro‐caspase‐3, PI3K, Akt, and upregulation of caspase‐8 and Bax.^151^ In a mouse xenograft model, the gold‐resveratrol nanoparticles reduced the tumor growth by decreasing the expression of vascular endothelial growth factor (VEGF) and promoting apoptosis in tumor tissue. In addition, there was no observable organ toxicity in the heart, liver, kidney, and spleen as assessed by histological studies. Moreover, the gold nanoparticles effectively increased the uptake of resveratrol into cells and localized near mitochondria. Therefore, these nanoparticles possess significantly better antitumor efficacy than resveratrol alone, both in vitro and in vivo.^151^ Similarly, glycyrrhizic acid‐conjugated human serum albumin nanoparticles loaded with resveratrol for liver tumor targeting was prepared by high‐pressure homogenization. The particle size of 108 nm, PDI of 0.001, encapsulation efficiency of 83.6% were observed, and resveratrol in the nanoparticles was found to be in an amorphous state. The nanoparticles showed a sustained release pattern and 2‐fold higher cytotoxicity and greater cellular uptake as compared to free resveratrol. Moreover, the nanoparticles were labeled with near‐IR fluorophore Cy5 to monitor the in vivo body distribution of nanoparticles in H22 tumor‐bearing mice. The near‐IR fluorescence images showed an enhanced distribution of nanoparticles to the liver tumors and a sustained‐release pattern. This study demonstrates the influence of formulation parameters such as human serum albumin concentration, homogenization speed, pressure, duration, and water to the organic phase volume ratio on the particle size and drug loading efficiency. Moreover, this study also showed the beneficial effect of combining the drugs with albumin to prevent deposition of the drug at the injection site and obtain a slow release of the drug.^152^

### 4.6 Lung cancer

Lung cancer is the foremost of all cancer‐related deaths worldwide, with approximately 69% reported in developing countries.^14^ Current treatments include a combination of surgery, radiation therapy, and chemotherapy. Resveratrol has been reported to be very promising in suppressing lung cancer by the phosphorylation of ribosome binding protein and transcriptional factors such as NFkB, which is accompanied by the induction of p21WAF1/CIP and increased activity of caspase 3, inducing apoptosis.^153^ Whereas in human epidermoid A431 cells, resveratrol showed cell cycle arrest in the G1 phase in addition to the induction of p21/WAF1.^154^

#### 4.6.1 In vitro studies

In a study by Karthikeyan et al (2015), resveratrol‐loaded gelatin nanoparticles were prepared (by coacervation method) for effective intracellular delivery in H460 lung cancer cell lines. The nano‐resveratrol was shown to produce higher cellular uptake in H460 lung cancer cells, which was associated with greater DNA damage and apoptotic incidence as compared to resveratrol alone. The mechanism of apoptosis includes the downregulation of Bcl‐2 and NFkB expression and the upregulation of Bax, p53, p21, and caspase‐3 expression. This enhanced anticancer activity also induced the arrest of the G0/G1 phase of the cell cycle.^107^ In another study, liposomes modified with dequalinium‐PEG‐distearoylphosphatidylethanolamine to allow selective uptake of resveratrol by the liposomes were synthesized. The liposomes' particle size was very small (70 nm) for intracellular delivery and induced apoptosis in both nonresistant and resistant cancer cells and showed increased cellular uptake and selective accumulation of drugs within mitochondria. Resveratrol was found to exhibit significant antitumor activity in both the A549/cDDP tumor spheroids and in xenograft‐resistant A549/cDDP cancer in nude mice; therefore, the mitochondrial targeting approach is used as a combinatorial tool in chemotherapy. The mechanism for enhanced efficacy of mitochondria, targeting resveratrol liposomes, might be due to (a) smaller particle size (70 nm), allowing the accumulation of the drug at the tumor site by EPR effect (b) long circulatory effect of PEG preventing the escape of drug by the reticuloendothelial system. Despite its potential advantages as an effective chemotherapeutic agent, the mechanism for the enhanced mitochondrial uptake of liposomes is still unclear.^108^

#### 4.6.2 In vivo studies

In a study by Karthikeyan et al, resveratrol‐loaded gelatin nanoparticles were prepared, which showed a mean particle diameter of 294 nm, PDI of 0.295, zeta potential of −18.6 mV, and encapsulation efficiency of 93.6%. In vitro release kinetics showed a rapid burst release followed by sustained release of resveratrol from gelatin nanoparticles. They also showed a rapid and more efficient cellular uptake, greater antiproliferative efficacy, greater ROS generation, DNA damage, and apoptosis than the free resveratrol in NCI‐H460 cells. Moreover, the bioavailability and half‐life were higher with twice the serum levels of resveratrol with nanoparticles compared to the free resveratrol in Swiss albino mice.^109^, ^155^ In another study, lyotropic liquid crystalline nanoparticles (based on glyceryl monoolein) for co‐delivery of pemetrexed and resveratrol for lung cancer treatment were prepared by hydrotrope method. The nanoparticles exhibited a particle size of 173 nm with a biphasic release pattern and a sustained release up to 24 hours. Also, the nanoparticles manifested a superior cytotoxicity profile against A549 lung cancer cells compared to free drug. The cellular uptake was also enhanced due to the bioadhesive properties of glyceryl monoolein. In vivo evaluations showed effective inhibition of tumor growth by inhibiting angiogenesis and inducing apoptosis in urethane‐induced lung cancer‐bearing mice. Therefore, these nanoparticles are proved to be a promising delivery carrier with no toxicity.^93^ In another study by Song et al (2018), epidermal growth factor conjugated core‐shell lipid‐polymer hybrid nanoparticles were fabricated to co‐deliver docetaxel and resveratrol.^14^ The in vitro and in vivo data show that the nanoparticles have significant synergistic cytotoxic effects, best tumor inhibition ability in mice, lowest systemic toxicity as seen from higher concentration at the target site of the lungs (at 48 hours post‐dose), and lower levels in the other organs (heart and kidney) compared to the free drugs (docetaxel/resveratrol).^14^ In another study by Huang et al (2018), ultrathin rhenium disulfide (UtRes_2_) nanosheet was prepared through the bovine serum albumin assisted ultrasonic exfoliation method.^156^ Resveratrol was loaded onto the nanosheet surface, and folic acid is then conjugated to the nanocomposite. A high loading ratio was achieved due to the presence of albumin with a large surface area. The nanocomposites released 16.5% of resveratrol over 24 hours and reached about 55% in 6 cycles of NIR irradiation. It also showed low cytotoxicity and excellent targeting effect in HepG2 cells. Upon pH/temperature dual‐stimuli, the targeted nanocomposite system showed an enhanced cytotoxic effect. Moreover, the targeted nanocomposite preparation intravenously injected into tumor‐bearing mice showed active targeting and greater accumulation in tumor tissue at 24 hours post‐injection. This was followed by effective tumor suppression without relapse after 30 days, when the injection was accompanied by 3 cycles of near‐infrared irradiation for 5 minutes, once a day. These findings suggested that the targeted nanocomposite system has a remarkable targeting ability, providing a dual‐stimuli‐responsive drug delivery system. This high loading efficiency might be due to large surface area of UtRes_2_, allowing the binding of functional groups through non‐covalent interactions such as hydrophobic or π‐π stacking. However, the higher stability of nanosheets was due to the adhesion of bovine serum albumin and PEG conjugation onto the nanosheet surface. The release of resveratrol was higher in the weak acidic environment of the tumor. In the acidic environment, protons are released, altering the hydrophilic/hydrophobic balance of the nanoparticles. Further studies revealed the significance of folic acid conjugation to promote cell internalization through receptor‐mediated endocytosis, thus providing an enhanced cytotoxic effect.^156^ Similarly, in another study by Geng et al (2017), resveratrol‐loaded human serum albumin nanoparticles conjugating RGD (arginine‐glycine‐aspartate) via a PEG “bridge” was prepared for effective targeted tumor therapy.^157^ The nanoparticles had an average diameter of 120 nm, encapsulation efficiency of 62.5%, and a maximum release ratio of 58.4% at pH 5.0. Confocal fluorescence images showed that the nanoparticles have the highest cellular uptake ratio (47.3%), attributing to an RGD‐mediated effect. The nanoparticles without resveratrol showed limited cytotoxicity, and the loaded nanoparticles were significantly more cytotoxic to PANC‐1 cells compared to free resveratrol. Moreover, the coating of nanoparticles with PEG and human serum albumin prolonged the blood circulation of resveratrol, increasing the half‐life approximately 5‐fold (1.2 hours for free resveratrol vs 6.6 hours for the nanoparticles). Similarly, after i.v administration, the tumor tissue was increased by 3‐ and 8‐fold respectively, compared to unconjugated and free resveratrol, respectively. These findings suggest that the conjugated nanoparticles have best tumor suppression ability with improved biocompatibility and prolonged circulation with no significant toxicity.^157^

### 4.7 Ovarian cancer

Ovarian cancer is the primary cause of cancer‐related deaths among all gynecological cancers, and chemotherapy is the most common treatment in many cases. However, if the tumor is well‐differentiated and confined to the ovary, surgery is the first choice of treatment. The anticancer activity of resveratrol was due to inhibition of STAT3 signaling.^158^ The role of autophagy was also found in resveratrol‐induced apoptotic cell death in OVCAR‐3 and CAOV‐3 human ovarian cancer cells. Resveratrol causes the generation of ROS, causing autophagy and subsequent apoptosis.^159^ Resveratrol is shown to lower the glucose uptake significantly and levels of phosphorylated Akt and mTOR in epithelial ovarian cancer cells.^160^

#### 4.7.1 In vitro studies

The chemotherapeutic activity of resveratrol‐loaded bovine serum albumin nanoparticles in human SKOV3 ovarian cancer cell lines was investigated. The nanoparticles induced apoptosis in a dose‐dependent manner, and the translocation of apoptosis‐inducing factor (AIF) from mitochondria to cytoplasm occurred even before the Cyto C. Moreover, binding of Bax to the mitochondria was essential for the release of AIF and Cyto C. Thus, it was concluded that resveratrol‐loaded bovine serum albumin nanoparticles induced apoptosis through AIF apoptosis pathway, which is considered as an alternative to the caspase‐dependent apoptosis pathway. This is the first study to investigate the mechanism for caspase‐independent apoptotic pathway. While AIF protein is an important factor in caspase‐independent pathway, further research is required to understand the mechanism by which AIF causes DNA ladder formation and also the association between early response signal and apoptotic signal.^110^ In another study, resveratrol‐loaded herbal extract (Angelica Gigas Nakai) (AGN) based nanoparticles were prepared using the nanocrystal concept.^161^ Nanoparticles are converted from crystalline to amorphous states by emulsification and solvent evaporation methods. The nanoparticles showed a particle size of 224 nm and negative zeta potential values. Sustained‐release profiles (for 5 days) were observed for decursin, decursinol angelate (the representative markers of the herbal extract), and resveratrol at pH 7.4. Even though the nanoparticles showed a lower cellular entry rate than AGN nanoparticles, the accumulated amount of nanoparticles in the cells was similar to that of AGN nanoparticles. The anti‐proliferation efficiency of the resveratrol nanoparticle in SKOV‐3 cells was significantly higher than the AGN extract, AGN nanoparticles, and AGN nanoparticles with resveratrol. The higher antiproliferative efficacy via endocytosis suggests the effective drug loading strategy of AGN extract and resveratrol in a single nanocarrier system. Moreover, it is important to limit the number of pharmaceutical excipients in the formulation, which could limit its toxicity after intravenous administration. This study utilized diverse ingredients to prepare nanoparticles, thereby eliminating the need for other pharmaceutical excipients except for a small amount of stabilizer. It also greatly reduced the toxicity, thereby increasing the feasibility of nanoparticles for clinical applications.^161^

#### 4.7.2 In vivo studies

The co‐delivery systems of micellar resveratrol‐quercetin or micellar resveratrol‐curcumin nanoparticles for Adriamycin (ADR) were reported. The efficacy of ADR was improved while mitigating its cardiotoxicity by administering a combination of micellar resveratrol‐quercetin or micellar resveratrol‐curcumin in healthy mice and ovarian cancer xenograft bearing mice. The results indicate that micellar resveratrol‐quercetin and ADR showed a significantly greater tumor size reduction in the xenograft model than ADR alone. Moreover, the left ventricular ejection fraction and fractional shortening in the ADR‐treated group is highly compromised in healthy mice. Therefore, coadministration of micellar resveratrol‐quercetin with ADR could possibly lead to a reduction of the dose while being cardioprotective. Based on these results, it is important to focus on the exact mechanism of cardiotoxicity in future studies in order to develop potential strategies to treat ADR‐induced cardiotoxicity.^94^ In another study, zinc oxide (ZnO) nanoparticles conjugated with trans‐resveratrol has been designed as a potential drug for ovarian cancer treatment. The nano‐conjugate was found to be more cytotoxic in comparison to free resveratrol. In vivo studies also demonstrated a considerable enhancement of antioxidant property (through ROS) and mitochondrial membrane depolarization in resveratrol‐loaded zinc oxide nano‐conjugate compared to free resveratrol. From the results, it appears that the smaller particle size was a factor in interacting with the cells, and the transfer of electrons from ZnO to resveratrol might be responsible for the increase in the ROS activity. Therefore, resveratrol‐loaded ZnO nano‐conjugate can be considered as a potential targeted drug delivery system.^111^

## 5 CURRENT CHALLENGES AND FUTURE PERSPECTIVE

Among several phytochemicals, phytoestrogens, such as stilbenes, particularly resveratrol, have been reported to have beneficial effects on human health with antioxidant, anti‐inflammatory, cardioprotective, anticarcinogenic, antiestrogenic, and neuroprotective properties.^162^ Due to the complexity in cancer cell signaling, the therapeutic benefit of specific inhibitors that can target only one network is limited. However, resveratrol is considered as a potent anticancer agent because it has both chemopreventive and chemotherapeutic effects by targeting multiple molecular pathways.^163^ Moreover, resveratrol affects all three stages (initiation, promotion, and progression) of carcinogenesis, and it is shown to induce the apoptotic pathway through several mechanisms.^164^ In addition to its anticarcinogenic activity as a single agent, its coadministration with other chemotherapeutic agents could reduce the associated side effects while enhancing the therapeutic efficacy in cancer chemotherapy. Resveratrol was combined with 5‐fluorouracil (skin cancer), curcumin, paclitaxel or exemestane (breast cancer), curcumin or docetaxel (prostate cancer), gold (liver cancer), docetaxel (lung cancer), quercetin, Adriamycin (ovarian cancer), either to reduce the chemotoxicity or to potentiate the effect of chemotherapeutic drugs by the combination therapy.

Despite its wider pharmacological activities, including anticancer activity, resveratrol poses pharmacokinetic challenges leading to poor bioavailability. Nanotechnology has revolutionized the delivery of several polyphenolic compounds such as resveratrol by surpassing various physicochemical, biochemical, and metabolization barriers. This review provided comprehensive information on various nanoparticle‐based delivery approaches to enhance the solubility, encapsulation efficiency, and anticancer activity of resveratrol. However, certain limitations of nanoparticles need to be addressed, such as nanomaterials toxicity, stability, and poor drug loading capacity of the nanoformulations. In a study by Jose et al (2014), the formulations showed low encapsulation efficiency ranging from 0.6% to 3.1%. The formulation with tween 80 alone had the lowest encapsulation efficiency and increased when two or more surfactants were utilized. This is due to enhanced solubilization of resveratrol in the combination of surfactants. Therefore, it is important to consider the formulation parameters such as surfactant composition and the drug‐lipid ratio in order to achieve higher encapsulation of the drug in the lipid core.^165^ In contrast, conventional polymeric micelles have reasonable encapsulation efficiency; however, they have poor stability in vivo. This is due to the physical assembly of conventional polymeric micelles leading to micelle disintegration. Therefore, it is important to consider alternatives such as disulfide‐mediated crosslinked micelles, which can be reversely disintegrated by reducing agents^166^ or to generate unimolecular micelles, constructed by coupling of L‐lactide with mPEG, thus forming a highly stable unimolecular micelle.^167^ Likewise, polymeric nanoparticles had good drug loading efficiency and stability; however, scale‐up of the current synthetic technique is a major challenge hindering its commercial availability.^168^ Moreover, there is a limited number of safe and biocompatible polymers available for the preparation of nanoparticles. All these challenges can be addressed by exploring various natural and biocompatible polymers (lignin, albumin) for the preparation of nanoparticles, utilizing novel conjugating ligands to enhance the cellular uptake and internalization, employing various computational and mathematical models to better predict the transportation into and through the tumors and improving the in vivo testing to establish the toxicity profile of the nanoparticles. Apart from challenges posed by various drug delivery approaches, nanoparticles as such poses various limitations such as complex and expensive synthetic techniques and the need to incorporate toxic reagents for its chemical synthesis, which might pose various environmental problems. Moreover, various ethical and regulatory requirements should be met to completely evaluate the long‐term effects on the environment, humans, and animals. Thus, it is necessary to perform post‐marketing surveillance even after FDA approval.^169^

Despite the large number of preclinical studies focused on chemopreventive effects of resveratrol nanoformulations, its transition to the clinic is far from reality due to various limitations. There is only limited clinical evidence of resveratrol as an effective supplement for cancer treatment and prevention. The first phase I clinical trial of resveratrol in colorectal cancer patients (n = 8) showed that resveratrol, when combined with other anticancer agents, could reduce the risk of colon cancer by decreasing Wnt target gene expression. One of the limitations of this trial is the detection of a significantly lower amount of resveratrol by HPLC analysis. Thus, this trial highlighted the importance of choosing the right formulation and dose for future studies.^170^ In the second clinical trial, supplementation of micronized resveratrol, formulated as a suspension (5 g/day for 10‐21 days, n = 6), resulted in increased caspase‐3 expression, thereby increasing apoptosis in cancerous cells compared to the placebo group. Thus, this trial suggested the administration of higher doses to achieve significant apoptosis induction.^171^ Other clinical studies focused on supplementation of resveratrol (2.5 g/day for 29 days) for reducing the levels of IGF‐1 and IGFBP3.^172^ Overall, all these clinical trials had a very small patient sample size, highlighting the scarcity of human data for resveratrol, thus focusing on the need for more research into the safety and efficacy of resveratrol. In addition to the completed trials, some of the ongoing trials focused on determining the optimal dose of resveratrol formulation that will result in effective plasma levels (NCT00433576) in colon cancer (ClinicalTrials.gov). This is important because the optimal dose of resveratrol is yet to be established. All these trials provide us with key suggestions for future clinical studies to make the most effective treatment regimens. Based on the analysis of these clinical trials, it is essential to focus on (a) the exact mechanism of action of resveratrol, (b) establishing the optimal dose, and (c) developing novel formulations of resveratrol with other agents. Considering all these parameters in the future will likely result in bringing the resveratrol nanoformulations from bench to bedside.

## 6 CONCLUSION

To overcome the extreme heterogeneity of cancer cells, an ideal therapeutic agent should target multiple biochemical pathways while limiting toxicity to healthy tissues. Among the naturally occurring anticancer agents, resveratrol has the potential to be an effective chemopreventive agent due to its ability to interact with several molecular targets involved in carcinogen metabolism, cell proliferation, apoptosis, etc. Resveratrol was also shown to modulate various signal transduction pathways. Despite being a potential chemopreventive agent, resveratrol presents various limitations such as low chemical stability and poor absorption and tumor delivery. In order to effectively overcome these shortcomings and to improve the bioavailability and cellular uptake of resveratrol, novel nanostructured delivery systems have been recently evolved. Various nanotechnology approaches were attempted for resveratrol for its beneficial effects in cancer chemoprevention as well as therapy. Despite these challenges in nanoformulations, the authors foresee a growth in the resveratrol nanomedicines in the coming years, due to the advancements in nanoengineering and bioengineering methods.

## CONFLICT OF INTEREST

The authors have no conflict of interest with any parties with the contents in this manuscript.

## AUTHORS' CONTRIBUTIONS

All authors had full access to the data in the study and take responsibility for the integrity of the data and the accuracy of the data analysis. *Data Curation*, M.A., I.P., A.T., R.J.B.; *Conceptualization*, S.H.S.B, R.D.A., R.J.B.; *Methodology*, I.P., R.D.A.; *Formal Analysis*, S.H.S.B, R.D.A., A.K., R.J.B.; *Writing‐Original Draft*, M.A., I.P., R.D.A; *Writing‐Review & Editing*, M.A., S.H.S.B, R.D.A., A.T., R.J.B.; *Visualization*, R.D.A., R.J.B.; *Validation*, A.K.; *Funding Acquisition*, R.J.B.; *Project Administration*, R.J.B.; *Supervision*, R.J.B.

## ETHICAL STATEMENT

Not Applicable.

## Data Availability

Data sharing not applicable to this article as no datasets were generated or analysed during the current study.

## References

[1] Cragg GM , Newman DJ . Natural products: a continuing source of novel drug leads. Biochim Biophys Acta. 2013;1830(6):3670‐3695. 23428572 10.1016/j.bbagen.2013.02.008 PMC3672862

[2] Howes M‐JR , Simmonds MSJ . The role of phytochemicals as micronutrients in health and disease. Curr Opin Clin Nutr Metab Care. 2014;17(6):558‐566. 25252018 10.1097/MCO.0000000000000115

[3] Mh C , Ringel BL . Does diet or alcohol explain the French paradox. Lancet. 1994;344:1719‐1723. 7996999 10.1016/s0140-6736(94)92883-5

[4] Soleas GJ , Grass L , Josephy PD , Goldberg DM , Diamandis EP . A comparison of the anticarcinogenic properties of four red wine polyphenols. Clin Biochem. 2002;35(2):119‐124. 11983346 10.1016/s0009-9120(02)00275-8

[5] Soleas GJ , Yan J , Goldberg DM . Ultrasensitive assay for three polyphenols (catechin, quercetin and resveratrol) and their conjugates in biological fluids utilizing gas chromatography with mass selective detection. J Chromatogr B Biomed Sci Appl. 2001;757(1):161‐172. 11419741 10.1016/s0378-4347(01)00142-6

[6] Park E‐J , Pezzuto JM . The pharmacology of resveratrol in animals and humans. BBA‐Mol Basis Dis. 2015;1852(6):1071‐1113. 10.1016/j.bbadis.2015.01.014 25652123

[7] Clouser CL , Chauhan J , Bess MA , et al. Anti‐HIV‐1 activity of resveratrol derivatives and synergistic inhibition of HIV‐1 by the combination of resveratrol and decitabine. Bioorg Med Chem Lett. 2012;22(21):6642‐6646. 23010273 10.1016/j.bmcl.2012.08.108 PMC3482103

[8] Abba Y , Hassim H , Hamzah H , Noordin MM . Antiviral activity of resveratrol against human and animal viruses. Adv Virol. 2015;2015:1‐7. 10.1155/2015/184241 PMC4676993 26693226

[9] Heredia A , Davis C , Redfield R . Synergistic inhibition of HIV‐1 in activated and resting peripheral blood mononuclear cells, monocyte‐derived macrophages, and selected drug‐resistant isolates with nucleoside analogues combined with a natural product, resveratrol. J Acquir Immune Defic Syndr Hum Retrovirol. 2000;25(3):246‐255. 10.1097/00126334-200011010-00006 11115955

[10] Jang M , Cai L , Udeani GO , et al. Cancer chemopreventive activity of resveratrol, a natural product derived from grapes. Science. 1997;275(5297):218‐220. 8985016 10.1126/science.275.5297.218

[11] World Health Organization . Cancer. 2020; https://www.who.int/health-topics/cancer#tab=tab_1. Accessed July 07.

[12] Elzoghby AO , El‐Lakany SA , Helmy MW , Abu‐Serie MM , Elgindy NA . Shell‐crosslinked zein nanocapsules for oral codelivery of exemestane and resveratrol in breast cancer therapy. Nanomedicine. 2017;12(24):2785‐2805. 29094642 10.2217/nnm-2017-0247

[13] Singh SK , Lillard JW Jr , Singh R . Reversal of drug resistance by planetary ball milled (PBM) nanoparticle loaded with resveratrol and docetaxel in prostate cancer. Cancer Lett. 2018;427:49‐62. 29678549 10.1016/j.canlet.2018.04.017 PMC5953846

[14] Song Z , Shi Y , Han Q , Dai G . Endothelial growth factor receptor‐targeted and reactive oxygen species‐responsive lung cancer therapy by docetaxel and resveratrol encapsulated lipid‐polymer hybrid nanoparticles. Biomed Pharmacother. 2018;105:18‐26. 29843041 10.1016/j.biopha.2018.05.095

[15] Rossi D , Guerrini A , Bruni R , et al. Trans‐resveratrol in nutraceuticals: issues in retail quality and effectiveness. Molecules. 2012;17(10):12393–12405. 23090020 10.3390/molecules171012393 PMC6268383

[16] Bertrand N , Wu J , Xu X , Kamaly N , Farokhzad OC . Cancer nanotechnology: the impact of passive and active targeting in the era of modern cancer biology. Adv Drug Del Rev. 2014;66:2‐25. 10.1016/j.addr.2013.11.009 PMC4219254 24270007

[17] Robinson K , Mock C , Liang D . Pre‐formulation studies of resveratrol. Drug Dev Ind Pharm. 2015;41(9):1464‐1469. 25224342 10.3109/03639045.2014.958753 PMC4427559

[18] Amri A , Chaumeil JC , Sfar S , Charrueau C . Administration of resveratrol: what formulation solutions to bioavailability limitations? J Control Release. 2012;158(2):182‐193. 21978644 10.1016/j.jconrel.2011.09.083

[19] Perrone D , Fuggetta MP , Ardito F , et al. Resveratrol (3, 5, 4′‐trihydroxystilbene) and its properties in oral diseases. Exp Ther Med. 2017;14(1):3‐9. 28672886 10.3892/etm.2017.4472 PMC5488484

[20] Soleas GJ , Diamandis EP , Goldberg DM . Resveratrol: a molecule whose time has come? And gone? Clin Biochem. 1997;30(2):91‐113. 9127691 10.1016/s0009-9120(96)00155-5

[21] Trela BC , Waterhouse AL . Resveratrol: isomeric molar absorptivities and stability. J Agric Food Chem. 1996;44(5):1253‐1257.

[22] Deak M , Falk H . On the chemistry of the resveratrol diastereomers. Monatsh Chem. 2003;134(6):883‐888.

[23] Walle T . Bioavailability of resveratrol. Ann N Y Acad Sci. 2011;1215(1):9‐15. 21261636 10.1111/j.1749-6632.2010.05842.x

[24] Albuquerque B , Costa MS , Peca IN , Cardoso MM . Production of double‐walled nanoparticles containing meloxicam. Polym Eng Sci. 2013;53(1):146‐152.

[25] Sergides C , Chirilă M , Silvestro L , Pitta D , Pittas A . Bioavailability and safety study of resveratrol 500 mg tablets in healthy male and female volunteers. Exp Ther Med. 2016;11(1):164‐170. 26889234 10.3892/etm.2015.2895 PMC4726856

[26] Singh G , Pai RS . Optimized PLGA nanoparticle platform for orally dosed trans‐resveratrol with enhanced bioavailability potential. Expert Opin Drug Deliv. 2014;11(5):647‐659. 24661109 10.1517/17425247.2014.890588

[27] Wenzel E , Somoza V . Metabolism and bioavailability of trans‐resveratrol. Mol Nutr Food Res. 2005;49(5):472‐481. 15779070 10.1002/mnfr.200500010

[28] Walle T , Hsieh F , DeLegge MH , Oatis JE , Walle UK . High absorption but very low bioavailability of oral resveratrol in humans. Drug Metab Dispos. 2004;32(12):1377‐1382. 15333514 10.1124/dmd.104.000885

[29] Vijayakumar MR , Kumari L , Patel KK , et al. Intravenous administration of trans‐resveratrol‐loaded TPGS‐coated solid lipid nanoparticles for prolonged systemic circulation, passive brain targeting and improved in vitro cytotoxicity against C6 glioma cell lines. RSC Adv. 2016;6(55):50336–50348.

[30] Wang P , Sang S . Metabolism and pharmacokinetics of resveratrol and pterostilbene. Biofactors. 2018;44(1):16‐25. 29315886 10.1002/biof.1410

[31] Balasubramanian SV , Straubinger RM . Taxol‐lipid interactions: taxol‐dependent effects on the physical properties of model membranes. Biochemistry (Mosc). 1994;33(30):8941‐8947. 10.1021/bi00196a011 7913831

[32] Chen L , Alrbyawi H , Poudel I , Arnold RD , Babu RJ . Co‐delivery of doxorubicin and Ceramide in a liposomal formulation enhances cytotoxicity in murine B16BL6 melanoma cell lines. AAPS PharmSciTech. 2019;20(3):99. 30719596 10.1208/s12249-019-1316-0

[33] Zhu G , Mock JN , Aljuffali I , Cummings BS , Arnold RD . Secretory phospholipase A2 responsive liposomes. J Pharm Sci. 2011;100(8):3146‐3159. 21455978 10.1002/jps.22530 PMC3196631

[34] Missaoui WN , Arnold RD , Cummings BS . Toxicological status of nanoparticles: what we know and what we don't know. Chem Biol Interact. 2018;295:1‐12. 30048623 10.1016/j.cbi.2018.07.015 PMC8427273

[35] Kang JY , Eggert M , Mouli S , et al. Pharmacokinetics, antitumor and cardioprotective effects of liposome‐encapsulated phenylaminoethyl selenide in human prostate cancer rodent models. Pharm Res. 2015;32(3):852‐862. 25158648 10.1007/s11095-014-1501-5 PMC4329269

[36] Bae YH , Park K . Targeted drug delivery to tumors: myths, reality and possibility. J Control Release. 2011;153(3):198‐205. 21663778 10.1016/j.jconrel.2011.06.001 PMC3272876

[37] Singh R , Lillard JW Jr . Nanoparticle‐based targeted drug delivery. Exp Mol Pathol. 2009;86(3):215‐223. 19186176 10.1016/j.yexmp.2008.12.004 PMC3249419

[38] Plapied L , Duhem N , des Rieux A , Préat V . Fate of polymeric nanocarriers for oral drug delivery. Curr Opin Colloid Interface Sci. 2011;16(3):228‐237.

[39] Deckert P . Current constructs and targets in clinical development for antibody‐based cancer therapy. Curr Drug Targets. 2009;10(2):158‐175. 19199912 10.2174/138945009787354502

[40] Seruga B , Ocana A , Tannock IF . Drug resistance in metastatic castration‐resistant prostate cancer. Nat Rev Clin Oncol. 2011;8(1):12‐23. 20859283 10.1038/nrclinonc.2010.136

[41] Banerjee S , Bueso‐Ramos C , Aggarwal BB . Suppression of 7, 12‐dimethylbenz (a) anthracene‐induced mammary carcinogenesis in rats by resveratrol: role of nuclear factor‐κB, cyclooxygenase 2, and matrix metalloprotease 9. Cancer Res. 2002;62(17):4945‐4954. 12208745

[42] Touzet O , Philips A . Resveratrol protects against protease inhibitor‐induced reactive oxygen species production, reticulum stress and lipid raft perturbation. J AIDS Clin Res. 2010;24(10):1437‐1447. 10.1097/QAD.0b013e32833a6114 20539089

[43] Sinha R , Kim GJ , Nie S , Shin DM . Nanotechnology in cancer therapeutics: bioconjugated nanoparticles for drug delivery. Mol Cancer Ther. 2006;5(8):1909‐1917. 16928810 10.1158/1535-7163.MCT-06-0141

[44] Hall JB , Dobrovolskaia MA , Patri AK , McNeil SE . Characterization of nanoparticles for therapeutics. Nanomedicine. 2007;2:789‐803. 18095846 10.2217/17435889.2.6.789

[45] Karve S , Werner ME , Sukumar R , et al. Revival of the abandoned therapeutic wortmannin by nanoparticle drug delivery. PNAS. 2012;109(21):8230‐8235. 22547809 10.1073/pnas.1120508109 PMC3361429

[46] Sanna V , Pala N , Sechi M . Targeted therapy using nanotechnology: focus on cancer. Int J Nanomed. 2014;9:467. 10.2147/IJN.S36654 PMC3896284 24531078

[47] Blanco E , Hsiao A , Mann AP , Landry MG , Meric‐Bernstam F , Ferrari M . Nanomedicine in cancer therapy: innovative trends and prospects. Cancer Sci. 2011;102(7):1247‐1252. 21447010 10.1111/j.1349-7006.2011.01941.x PMC11158341

[48] Sharma A , Sharma US . Liposomes in drug delivery: progress and limitations. Int J Pharm. 1997;154(2):123‐140.

[49] Coimbra M , Isacchi B , van Bloois L , et al. Improving solubility and chemical stability of natural compounds for medicinal use by incorporation into liposomes. Int J Pharm. 2011;416(2):433‐442. 21291975 10.1016/j.ijpharm.2011.01.056

[50] Sanna V , Siddiqui IA , Sechi M , Mukhtar H . Resveratrol‐loaded nanoparticles based on poly (epsilon‐caprolactone) and poly (d, l‐lactic‐co‐glycolic acid)–poly (ethylene glycol) blend for prostate cancer treatment. Mol Pharm. 2013;10(10):3871‐3881. 23968375 10.1021/mp400342f PMC4100701

[51] Souto EB , Müller RH . Lipid nanoparticles: effect on bioavailability and pharmacokinetic changes. In: Schäfer‐Korting M . (eds). Drug Delivery. Handbook of Experimental Pharmacology Springer, Berlin, Heidelberg; 2010;197:115‐141. 10.1007/978-3-642-00477-3_4 20217528

[52] Teskač K , Kristl J . The evidence for solid lipid nanoparticles mediated cell uptake of resveratrol. Int J Pharm. 2010;390(1):61‐69. 19833178 10.1016/j.ijpharm.2009.10.011

[53] Tiwari G , Tiwari R , Rai AK . Cyclodextrins in delivery systems: applications. J Pharm Bioallied Sci. 2010;2(2):72‐79. 21814436 10.4103/0975-7406.67003 PMC3147107

[54] Venuti V , Cannavà C , Cristiano MC , et al. A characterization study of resveratrol/sulfobutyl ether‐β‐cyclodextrin inclusion complex and in vitro anticancer activity. Colloids Surf B Biointerfaces. 2014;115:22‐28. 24321846 10.1016/j.colsurfb.2013.11.025

[55] Minchinton AI , Tannock IF . Drug penetration in solid tumours. Nat Rev Cancer. 2006;6(8):583‐592. 16862189 10.1038/nrc1893

[56] Zhang X‐Q , Xu X , Bertrand N , Pridgen E , Swami A , Farokhzad OC . Interactions of nanomaterials and biological systems: implications to personalized nanomedicine. Adv Drug Del Rev. 2012;64(13):1363‐1384. 10.1016/j.addr.2012.08.005 PMC3517211 22917779

[57] Maeda H . Macromolecular therapeutics in cancer treatment: the EPR effect and beyond. J Control Release. 2012;164(2):138‐144. 22595146 10.1016/j.jconrel.2012.04.038

[58] Wilhelm S , Tavares AJ , Dai Q , et al. Analysis of nanoparticle delivery to tumours. Nat Rev Mater. 2016;1(5):16014.

[59] Du J‐Z , Du X‐J , Mao C‐Q , Wang J . Tailor‐made dual pH‐sensitive polymer–doxorubicin nanoparticles for efficient anticancer drug delivery. J Am Chem Soc. 2011;133(44):17560–17563. 21985458 10.1021/ja207150n

[60] Hu C‐MJ , Zhang L . Therapeutic nanoparticles to combat cancer drug resistance. Curr Drug Metab. 2009;10(8):836‐841. 20214578 10.2174/138920009790274540

[61] Huwyler J , Cerletti A , Fricker G , Eberle AN , Drewe J . By‐passing of P‐glycoprotein using immunoliposomes. J Drug Target. 2002;10(1):73‐79. 11996089 10.1080/10611860290007559

[62] Kelloff GJ , Lieberman R , Steele VE , et al. Agents, biomarkers, and cohorts for chemopreventive agent development in prostate cancer. J Urol. 2001;57(4):46‐51. 10.1016/s0090-4295(00)00940-7 11295594

[63] Baur JA , Sinclair DAJND . Therapeutic potential of resveratrol: the in vivo evidence. Nat Rev Drug Discov. 2006;5(6):493‐506. 16732220 10.1038/nrd2060

[64] Saiko P , Szakmary A , Jaeger W , Szekeres T . Resveratrol and its analogs: defense against cancer, coronary disease and neurodegenerative maladies or just a fad? Mutat Res. 2008;658(1‐2):68‐94. 17890139 10.1016/j.mrrev.2007.08.004

[65] Aggarwal BB , Bhardwaj A , Aggarwal RS , Seeram NP , Shishodia S , Takada Y . Role of resveratrol in prevention and therapy of cancer: preclinical and clinical studies. Anticancer Res. 2004;24(5A):2783‐2840. 15517885

[66] Shankar S , Singh G , Srivastava RK . Chemoprevention by resveratrol: molecular mechanisms and therapeutic potential. Front Biosci. 2007;12(12):4839‐4854. 17569614 10.2741/2432

[67] Kundu JK , Surh Y‐J . Cancer chemopreventive and therapeutic potential of resveratrol: mechanistic perspectives. Cancer Lett. 2008;269(2):243‐261. 18550275 10.1016/j.canlet.2008.03.057

[68] Demoulin B , Hermant M , Castrogiovanni C , Staudt C , Dumont P . Resveratrol induces DNA damage in colon cancer cells by poisoning topoisomerase II and activates the ATM kinase to trigger p53‐dependent apoptosis. Toxicol in Vitro. 2015;29(5):1156‐1165. 25952326 10.1016/j.tiv.2015.04.015

[69] Saud SM , Li W , Morris NL , et al. Resveratrol prevents tumorigenesis in mouse model of Kras activated sporadic colorectal cancer by suppressing oncogenic Kras expression. Carcinogenesis. 2014;35(12):2778‐2786. 25280562 10.1093/carcin/bgu209 PMC4247523

[70] Pandey PR , Okuda H , Watabe M , et al. Resveratrol suppresses growth of cancer stem‐like cells by inhibiting fatty acid synthase. Breast Cancer Res Treat. 2011;130(2):387‐398. 21188630 10.1007/s10549-010-1300-6 PMC3404809

[71] Singh CK , George J , Ahmad N . Resveratrol‐based combinatorial strategies for cancer management. Ann N Y Acad Sci. 2013;1290(1):113‐121. 23855473 10.1111/nyas.12160 PMC3713511

[72] Apel K , Hirt H . Reactive oxygen species: metabolism, oxidative stress, and signal transduction. Annu Rev Plant Biol. 2004;55:373‐399. 15377225 10.1146/annurev.arplant.55.031903.141701

[73] Schieber M , Chandel NS . ROS function in redox signaling and oxidative stress. Curr Biol. 2014;24(10):R453‐R462. 24845678 10.1016/j.cub.2014.03.034 PMC4055301

[74] Nose K . Role of reactive oxygen species in the regulation of physiological functions. Biol Pharm Bull. 2000;23(8):897‐903. 10963291 10.1248/bpb.23.897

[75] Leonard SS , Xia C , Jiang B‐H , et al. Resveratrol scavenges reactive oxygen species and effects radical‐induced cellular responses. Biochem Biophys Res Commun. 2003;309(4):1017‐1026. 13679076 10.1016/j.bbrc.2003.08.105

[76] Granzotto A , Zatta P . Resveratrol acts not through anti‐aggregative pathways but mainly via its scavenging properties against Aβ and Aβ‐metal complexes toxicity. PLoS One. 2011;6(6):e21565. 21738712 10.1371/journal.pone.0021565 PMC3124535

[77] De La Lastra CA , Villegas I . Resveratrol as an antioxidant and pro‐oxidant agent: mechanisms and clinical implications. Biochemical Society Transactions. 2007;35(5):1156–1160. 17956300 10.1042/BST0351156

[78] Gülçin İ . Antioxidant properties of resveratrol: a structure–activity insight. Innov Food Sci Emerg Technol. 2010;11(1):210‐218.

[79] Rhayem Y , Thérond P , Camont L , et al. Chain‐breaking activity of resveratrol and piceatannol in a linoleate micellar model. Chem Phys Lipids. 2008;155(1):48‐56. 18590713 10.1016/j.chemphyslip.2008.06.001

[80] Mohan A , Narayanan S , Sethuraman S , Krishnan UM . Novel resveratrol and 5‐fluorouracil coencapsulated in PEGylated nanoliposomes improve chemotherapeutic efficacy of combination against head and neck squamous cell carcinoma. Biomed Res Int. 2014;2014:1‐14. 10.1155/2014/424239 PMC4119704 25114900

[81] Meng J , Guo F , Xu H , Liang W , Wang C , Yang X‐D . Combination therapy using co‐encapsulated resveratrol and paclitaxel in liposomes for drug resistance reversal in breast cancer cells in vivo. Sci Rep. 2016;6:22390. 26947928 10.1038/srep22390 PMC4780086

[82] Saralkar P , Dash AK . Alginate nanoparticles containing curcumin and resveratrol: preparation, characterization, and in vitro evaluation against DU145 prostate cancer cell line. AAPS PharmSciTech. 2017;18(7):2814‐2823. 28397161 10.1208/s12249-017-0772-7

[83] Gumireddy A , Christman R , Kumari D , Tiwari A , North EJ , Chauhan H . Preparation, characterization, and in vitro evaluation of Curcumin‐and resveratrol‐loaded solid lipid nanoparticles. AAPS PharmSciTech. 2019;20(4):145. 30887133 10.1208/s12249-019-1349-4

[84] El‐Far SW , Helmy MW , Khattab SN , Bekhit AA , Hussein AA , Elzoghby AO . Folate conjugated vs PEGylated phytosomal casein nanocarriers for codelivery of fungal‐and herbal‐derived anticancer drugs. Nanomedicine. 2018;13(12):1463‐1480. 29957120 10.2217/nnm-2018-0006

[85] Anwar DM , Khattab SN , Helmy MW , et al. Lactobionic/folate dual‐targeted amphiphilic maltodextrin‐based micelles for targeted codelivery of sulfasalazine and resveratrol to hepatocellular carcinoma. Bioconjug Chem. 2018;29(9):3026‐3041. 30110148 10.1021/acs.bioconjchem.8b00428

[86] Guo X , Zhao Z , Chen D , et al. Co‐delivery of resveratrol and docetaxel via polymeric micelles to improve the treatment of drug‐resistant tumors. Asian J Pharm Sci. 2019;14(1):78‐85. 32104440 10.1016/j.ajps.2018.03.002 PMC7032195

[87] Carlson LJ , Cote B , Alani AWG , Rao DA . Polymeric micellar co‐delivery of resveratrol and curcumin to mitigate in vitro doxorubicin‐induced cardiotoxicity. J Pharm Sci. 2014;103(8):2315‐2322. 24914015 10.1002/jps.24042

[88] Xu X , Liu A , Bai Y , et al. Co‐delivery of resveratrol and p53 gene via peptide cationic liposomal nanocarrier for the synergistic treatment of cervical cancer and breast cancer cells. J Drug Deliv Sci Technol. 2019;51:746‐753.

[89] Cosco D , Paolino D , Maiuolo J , et al. Ultradeformable liposomes as multidrug carrier of resveratrol and 5‐fluorouracil for their topical delivery. Int J Pharm. 2015;489(1–2):1‐10. 25899287 10.1016/j.ijpharm.2015.04.056

[90] Pushpalatha R , Selvamuthukumar S , Kilimozhi D . Cyclodextrin nanosponge based hydrogel for the transdermal co‐delivery of curcumin and resveratrol: development, optimization, in vitro and ex vivo evaluation. J Drug Deliv Sci Technol. 2019;52:55‐64.

[91] Hu Y , Wang Z , Qiu Y , Liu Y , Ding M , Zhang Y . Anti‐miRNA21 and resveratrol‐loaded polysaccharide‐based mesoporous silica nanoparticle for synergistic activity in gastric carcinoma. J Drug Target. 2019;27:1‐31. 31017473 10.1080/1061186X.2019.1610766

[92] Jaisamut P , Wiwattanawongsa K , Wiwattanapatapee R . A novel self‐microemulsifying system for the simultaneous delivery and enhanced oral absorption of curcumin and resveratrol. Planta Med. 2017;83(05):461‐467. 27280934 10.1055/s-0042-108734

[93] Abdelaziz HM , Elzoghby AO , Helmy MW , Samaha MW , Fang J‐Y , Freag MS . Liquid crystalline assembly for potential combinatorial chemo–herbal drug delivery to lung cancer cells. Int J Nanomedicine. 2019;14:499‐517. 30666110 10.2147/IJN.S188335 PMC6333390

[94] Al Fatease A , Shah V , Nguyen DX , et al. Chemosensitization and mitigation of Adriamycin‐induced cardiotoxicity using combinational polymeric micelles for co‐delivery of quercetin/resveratrol and resveratrol/curcumin in ovarian cancer. Nanomed Nanotechnol Biol Med. 2019;19:39‐48. 10.1016/j.nano.2019.03.011 31022465

[95] Wang W , Tang Q , Yu T , et al. Surfactant‐free preparation of au@ resveratrol hollow nanoparticles with photothermal performance and antioxidant activity. ACS Appl Mater Interfaces. 2017;9(4):3376‐3387. 28098974 10.1021/acsami.6b13911

[96] Carletto B , Berton J , Ferreira TN , et al. Resveratrol‐loaded nanocapsules inhibit murine melanoma tumor growth. Colloids Surf B Biointerfaces. 2016;144:65‐72. 27070053 10.1016/j.colsurfb.2016.04.001

[97] Park SY , Chae SY , Park JO , Lee KJ , Park G . Gold‐conjugated resveratrol nanoparticles attenuate the invasion and MMP‐9 and COX‐2 expression in breast cancer cells. Oncol Rep. 2016;35(6):3248‐3256. 27035791 10.3892/or.2016.4716

[98] Wang W , Zhang L , Chen T , et al. Anticancer effects of resveratrol‐loaded solid lipid nanoparticles on human breast cancer cells. Mol Cells. 2017;22(11):1814. 10.3390/molecules22111814 PMC6150230 29068422

[99] Abdel‐Latif GA , Al‐Abd AM , Tadros MG , Al‐Abbasi FA , Khalifa AE , Abdel‐Naim AB . The chemomodulatory effects of resveratrol and didox on herceptin cytotoxicity in breast cancer cell lines. Sci Rep. 2015;5:12054. 26156237 10.1038/srep12054 PMC4496837

[100] Nassir AM , Shahzad N , Ibrahim IA , Ahmad I , Md S , Ain MR . Resveratrol‐loaded PLGA nanoparticles mediated programmed cell death in prostate cancer cells. Saudi Pharm J. 2018;26(6):876‐885. 30202231 10.1016/j.jsps.2018.03.009 PMC6128707

[101] Chen P , Bai P , Luo G , et al. Role of anti‐apoptotic activity of antioxidants in conferring protection against prostate cancer. Int J Pharmacol. 2016;12(4):304‐316.

[102] Narayanan NK , Nargi D , Randolph C , Narayanan BA . Liposome encapsulation of curcumin and resveratrol in combination reduces prostate cancer incidence in PTEN knockout mice. Int J Cancer Prev. 2009;125(1):1‐8. 10.1002/ijc.24336 19326431

[103] Summerlin N , Qu Z , Pujara N , et al. Colloidal mesoporous silica nanoparticles enhance the biological activity of resveratrol. Colloids Surf B Biointerfaces. 2016;144:1‐7. 27060664 10.1016/j.colsurfb.2016.03.076

[104] Juère E , Florek J , Bouchoucha M , et al. In vitro dissolution, cellular membrane permeability, and anti‐inflammatory response of resveratrol‐encapsulated mesoporous silica nanoparticles. Mol Pharm. 2017;14(12):4431‐4441. 29094948 10.1021/acs.molpharmaceut.7b00529

[105] Jung K‐H , Lee JH , Park JW , et al. Resveratrol‐loaded polymeric nanoparticles suppress glucose metabolism and tumor growth in vitro and in vivo. Int J Pharm. 2015;478(1):251‐257. 25445992 10.1016/j.ijpharm.2014.11.049

[106] Wu J , Wang Y , Yang H , Liu X , Lu Z . Preparation and biological activity studies of resveratrol loaded ionically cross‐linked chitosan‐TPP nanoparticles. Carbohydr Polym. 2017;175:170‐177. 28917853 10.1016/j.carbpol.2017.07.058

[107] Karthikeyan S , Hoti SL , Prasad NR . Resveratrol loaded gelatin nanoparticles synergistically inhibits cell cycle progression and constitutive NF‐kappaB activation, and induces apoptosis in non‐small cell lung cancer cells. Biomed Pharmacother. 2015;70:274‐282. 25776512 10.1016/j.biopha.2015.02.006

[108] Wang X‐X , Li Y‐B , Yao H‐J , et al. The use of mitochondrial targeting resveratrol liposomes modified with a dequalinium polyethylene glycol‐distearoylphosphatidyl ethanolamine conjugate to induce apoptosis in resistant lung cancer cells. Biomaterials. 2011;32(24):5673‐5687. 21550109 10.1016/j.biomaterials.2011.04.029

[109] Karthikeyan S , Prasad N . Anticancer activity of resveratrol‐loaded gelatin nanoparticles on Ehrlich's ascites carcinoma in Swiss albino mice. Biomed Prev Nutr. 2013;1:31‐38.

[110] Guo L , Peng Y , Li Y , et al. Cell death pathway induced by resveratrol‐bovine serum albumin nanoparticles in a human ovarian cell line. Oncol Lett. 2015;9(3):1359‐1363. 25663913 10.3892/ol.2015.2851 PMC4315083

[111] Khatun M , Choudhury S , Liu B , Lemmens P , Pal SK , Mazumder SJRA . Resveratrol–ZnO nanohybrid enhanced anti‐cancerous effect in ovarian cancer cells through ROS. RSC Adv. 2016;6(107):105607‐105617.

[112] Rigon R , Fachinetti N , Severino P , Santana M , Chorilli M . Skin delivery and in vitro biological evaluation of trans‐resveratrol‐loaded solid lipid nanoparticles for skin disorder therapies. Mol Cells. 2016;21(1):116. 10.3390/molecules21010116 PMC6273087 26805794

[113] Linares MA , Zakaria A , Nizran P . Skin cancer. J Prim Care Community Health. 2015;42(4):645‐659. 10.1016/j.pop.2015.07.006 26612377

[114] Athar M , Back JH , Tang X , et al. Resveratrol: a review of preclinical studies for human cancer prevention. Toxicol Appl Pharmacol. 2007;224(3):274‐283. 17306316 10.1016/j.taap.2006.12.025 PMC2083123

[115] Kim A , Yang Y , Lee MS , Yoo YD , Lee HG , Lim JS . NDRG2 gene expression in B16F10 melanoma cells restrains melanogenesis via inhibition of Mitf expression. Pigment Cell Melanoma Res. 2008;21(6):653‐664. 19067970 10.1111/j.1755-148X.2008.00503.x

[116] Fontecave M , Lepoivre M , Elleingand E , Gerez C , Guittet O . Resveratrol, a remarkable inhibitor of ribonucleotide reductase. FEBS Lett. 1998;421(3):277‐279. 9468322 10.1016/s0014-5793(97)01572-x

[117] Fachinetti N , Rigon RB , Eloy JO , Sato MR , dos Santos KC , Chorilli M . Comparative study of glyceryl behenate or polyoxyethylene 40 stearate‐based lipid carriers for trans‐resveratrol delivery: development, characterization and evaluation of the in vitro tyrosinase inhibition. AAPS PharmSciTech. 2018;19(3):1401‐1409. 29404955 10.1208/s12249-018-0961-z

[118] Detoni CB , Souto GD , da Silva ALM , Pohlmann AR , Guterres SS . Photostability and skin penetration of different e‐resveratrol‐loaded supramolecular structures. Photochem Photobiol. 2012;88(4):913‐921. 22443373 10.1111/j.1751-1097.2012.01147.x

[119] Friedrich RB , Kann B , Coradini K , Offerhaus HL , Beck RC , Windbergs M . Skin penetration behavior of lipid‐core nanocapsules for simultaneous delivery of resveratrol and curcumin. Eur J Pharm Sci. 2015;78:204‐213. 26215463 10.1016/j.ejps.2015.07.018

[120] Afaq F , Adhami VM , Ahmad N . Prevention of short‐term ultraviolet B radiation‐mediated damages by resveratrol in SKH‐1 hairless mice. Toxicol Appl Pharmacol. 2003;186(1):28‐37. 12583990 10.1016/s0041-008x(02)00014-5

[121] Aziz MH , Afaq F , Ahmad N . Prevention of ultraviolet‐B radiation damage by resveratrol in mouse skin is mediated via modulation in Survivin¶. Photochem Photobiol. 2005;81(1):25‐31. 15469386 10.1562/2004-08-13-RA-274

[122] DeSantis C , Ma J , Bryan L , Jemal A . Breast cancer statistics, 2013. CA Cancer J Clin. 2014;64(1):52‐62. 24114568 10.3322/caac.21203

[123] Bertolini F . Adipose tissue and breast cancer progression: a link between metabolism and cancer. Breast. 2013;22:S48‐S49. 24074792 10.1016/j.breast.2013.07.009

[124] Song J , Su H , Zhou Y‐Y , Guo L‐L . Prognostic value of matrix metalloproteinase 9 expression in breast cancer patients: a meta‐analysis. Asian Pac J Cancer Prev. 2013;14(3):1615‐1621. 23679245 10.7314/apjcp.2013.14.3.1615

[125] Feng R , Song Z , Zhai G . Preparation and in vivo pharmacokinetics of curcumin‐loaded PCL‐PEG‐PCL triblock copolymeric nanoparticles. Int J Nanomed. 2012;7:4089. 10.2147/IJN.S33607 PMC3414087 22888245

[126] Le Corre L , Chalabi N , Delort L , Bignon YJ , Bernard‐Gallon D . Resveratrol and breast cancer chemoprevention: molecular mechanisms. Mol Nutr Food Res. 2005;49(5):462‐471. 15786518 10.1002/mnfr.200400094

[127] Basly J‐P , Marre‐Fournier F , Le Bail J‐C , Habrioux G , Chulia AJJL . Estrogenic/antiestrogenic and scavenging properties of (E)‐and (Z)‐resveratrol. Life Sci. 2000;66(9):769‐777. 10698352 10.1016/s0024-3205(99)00650-5

[128] Levenson AS , Gehm BD , Pearce ST , et al. Resveratrol acts as an estrogen receptor (ER) agonist in breast cancer cells stably transfected with ER α. Int J Canc Prev. 2003;104(5):587‐596. 10.1002/ijc.10992 12594813

[129] Kim S , Ng WK , Dong Y , Das S , Tan RB . Preparation and physicochemical characterization of trans‐resveratrol nanoparticles by temperature‐controlled antisolvent precipitation. J Food Eng. 2012;108(1):37‐42.

[130] Chakraborty S , Das T , Sarma HD , Venkatesh M , Banerjee S . Preparation and preliminary studies on 177Lu‐labeled hydroxyapatite particles for possible use in the therapy of liver cancer. Nucl Med Biol. 2008;35(5):589‐597. 18589303 10.1016/j.nucmedbio.2008.03.003

[131] Garvin S , Öllinger K , Dabrosin C . Resveratrol induces apoptosis and inhibits angiogenesis in human breast cancer xenografts in vivo. Cancer Lett. 2006;231(1):113‐122. 16356836 10.1016/j.canlet.2005.01.031

[132] Vergaro V , Lvov YM , Leporatti S . Halloysite clay nanotubes for resveratrol delivery to cancer cells. Macromol Biosci. 2012;12(9):1265‐1271. 22887783 10.1002/mabi.201200121

[133] Poonia N , Narang JK , Lather V , et al. Resveratrol loaded functionalized nanostructured lipid carriers for breast cancer targeting: systematic development, characterization and pharmacokinetic evaluation. Colloids Surf B Biointerfaces. 2019;181:756‐766. 31234063 10.1016/j.colsurfb.2019.06.004

[134] Hao J , Tong T , Jin K , et al. Folic acid‐functionalized drug delivery platform of resveratrol based on Pluronic 127/D‐α‐tocopheryl polyethylene glycol 1000 succinate mixed micelles. Int J Nanomed. 2017;12:2279‐2292. 10.2147/IJN.S130094 PMC5373843 28392687

[135] Jordan BC , Mock CD , Thilagavathi R , Selvam C . Molecular mechanisms of curcumin and its semisynthetic analogues in prostate cancer prevention and treatment. Life Sci. 2016;152:135‐144. 27018446 10.1016/j.lfs.2016.03.036 PMC4867249

[136] McMenamin ME , Soung P , Perera S , Kaplan I , Loda M , Sellers WR . Loss of PTEN expression in paraffin‐embedded primary prostate cancer correlates with high Gleason score and advanced stage. Cancer Res Treat. 1999;59(17):4291‐4296. 10485474

[137] Seeni A , Takahashi S , Takeshita K , et al. Suppression of prostate cancer growth by resveratrol in the transgenic rat for adenocarcinoma of prostate (TRAP) model. Asian Pac J Cancer Prev. 2008;9(1):7‐14. 18439064

[138] Sheth S , Jajoo S , Kaur T , et al. Resveratrol reduces prostate cancer growth and metastasis by inhibiting the Akt/MicroRNA‐21 pathway. PLoS One. 2012;7(12):e51655. 23272133 10.1371/journal.pone.0051655 PMC3521661

[139] Li Y , Eresen A , Lu Y , et al. Radiomics signature for the preoperative assessment of stage in advanced colon cancer. Am J Cancer Res. 2019;9(7):1429‐1438. 31392079 PMC6682712

[140] Mahadevappa R , Fai Kwok H . Phytochemicals‐a novel and prominent source of anti‐cancer drugs against colorectal cancer. Comb Chem High Throughput Screen. 2017;20(5):376‐394. 28078982 10.2174/1386207320666170112141833

[141] Namani A , Li J , Wang XJ , Tang XA . Review of compounds for prevention of colorectal cancer. Curr Pharmacol Rep. 2017;3(5):221‐231.

[142] Elshaer M , Chen Y , Wang XJ , Tang X . Resveratrol: an overview of its anti‐cancer mechanisms. Life sci. 2018;207:340–349. 29959028 10.1016/j.lfs.2018.06.028

[143] Arunachalam G , Yao H , Sundar IK , Caito S , Rahman I . SIRT1 regulates oxidant‐and cigarette smoke‐induced eNOS acetylation in endothelial cells: role of resveratrol. Biochem Biophys Res Commun. 2010;393(1):66‐72. 20102704 10.1016/j.bbrc.2010.01.080 PMC2830376

[144] Hope C , Planutis K , Planutiene M , et al. Low concentrations of resveratrol inhibit Wnt signal throughput in colon‐derived cells: implications for colon cancer prevention. Mol Nutr Food Res. 2008;52(S1):S52‐S61. 18504708 10.1002/mnfr.200700448 PMC2519107

[145] Sakoguchi‐Okada N , Takahashi‐Yanaga F , Fukada K , et al. Celecoxib inhibits the expression of survivin via the suppression of promoter activity in human colon cancer cells. Biochem Pharmacol. 2007;73(9):1318‐1329. 17270149 10.1016/j.bcp.2006.12.033

[146] Kamal R , Chadha VD , Dhawan D . Physiological uptake and retention of radiolabeled resveratrol loaded gold nanoparticles (99mTc‐res‐AuNP) in colon cancer tissue. Nanomed Nanotechnol Biol Med. 2018;14(3):1059‐1071. 10.1016/j.nano.2018.01.008 29391211

[147] Soo E , Thakur S , Qu Z , Jambhrunkar S , Parekh HS , Popat A . Enhancing delivery and cytotoxicity of resveratrol through a dual nanoencapsulation approach. J Colloid Interface Sci. 2016;462:368‐374. 26479200 10.1016/j.jcis.2015.10.022

[148] Cai Z , Liu Q . Understanding the global cancer statistics 2018: implications for cancer control. Sci China Life Sci. 2019;69:1‐4. 10.1007/s11427-019-9816-1 31463738

[149] Bu L , Gan L‐C , Guo X‐Q , et al. Trans‐resveratrol loaded chitosan nanoparticles modified with biotin and avidin to target hepatic carcinoma. Int J Pharm. 2013;452(1–2):355‐362. 23685116 10.1016/j.ijpharm.2013.05.007

[150] Luo S‐Q , Lu W‐L , Wang Y‐F , Wang Z‐P . Anti‐hepatocarcinoma effects of a food additive resveratrol Nanosuspension against human HepG2 cells. Adv J Food Sci Technol. 2015;8(3):210‐213.

[151] Zhang D , Zhang J , Zeng J , et al. Nano‐gold loaded with resveratrol enhance the anti‐hepatoma effect of resveratrol in vitro and in vivo. J Biomed Nanotechnol. 2019;15(2):288‐300. 30596551 10.1166/jbn.2019.2682

[152] Wu M , Lian B , Deng Y , et al. Resveratrol‐loaded glycyrrhizic acid‐conjugated human serum albumin nanoparticles wrapping resveratrol nanoparticles: preparation, characterization, and targeting effect on liver tumors. J Biomater Appl. 2017;32(2):191‐205. 28610486 10.1177/0885328217713357

[153] Kim Y , Lee WH , Choi TH , Rhee S‐H , Park K‐Y , Choi YH . Involvement of p21WAF1/CIP1, pRB, Bax and NF‐κB in induction of growth arrest and apoptosis by resveratrol in human lung carcinoma A549 cells. Int J Oncol. 2003;23(4):1143‐1149. 12963997

[154] Ahmad N , Adhami VM , Afaq F , Feyes DK , Mukhtar H . Resveratrol causes WAF‐1/p21‐mediated G1‐phase arrest of cell cycle and induction of apoptosis in human epidermoid carcinoma A431 cells. Clin Cancer Res. 2001;7(5):1466‐1473. 11350919

[155] Karthikeyan S , Prasad NR , Ganamani A , Balamurugan EJB , Nutrition P . Anticancer activity of resveratrol‐loaded gelatin nanoparticles on NCI‐H460 non‐small cell lung. Cancer Cells. 2013;3(1):64‐73.

[156] Huang Q , Wang S , Zhou J , Zhong X , YJRa H . Albumin‐assisted exfoliated ultrathin rhenium disulfide nanosheets as a tumor targeting and dual‐stimuli‐responsive drug delivery system for a combination chemo‐photothermal treatment. RSC Adv. 2018;8(9):4624‐4633. 10.1039/c7ra13454a PMC9077812 35539567

[157] Geng T , Zhao X , Ma M , Zhu G , Yin L . Resveratrol‐loaded albumin nanoparticles with prolonged blood circulation and improved biocompatibility for highly effective targeted pancreatic tumor therapy. J Nanoscale Res Lett. 2017;12(1):437. 10.1186/s11671-017-2206-6 PMC5493600 28673056

[158] Zhong L‐X , Li H , Wu M‐L , et al. Inhibition of STAT3 signaling as critical molecular event in resveratrol‐suppressed ovarian cancer cells. J Ovarian Res. 2015;8(1):25. 25896424 10.1186/s13048-015-0152-4 PMC4409989

[159] Lang F , Qin Z , Li F , Zhang H , Fang Z , Hao E . Apoptotic cell death induced by resveratrol is partially mediated by the autophagy pathway in human ovarian cancer cells. PLoS One. 2015;10(6):e0129196. 26067645 10.1371/journal.pone.0129196 PMC4466135

[160] Kueck A , Opipari AW Jr , Griffith KA , et al. Resveratrol inhibits glucose metabolism in human ovarian cancer cells. Gynecol Oncol. 2007;107(3):450‐457. 17825886 10.1016/j.ygyno.2007.07.065

[161] Nam S , Lee S , Kang W‐S , Cho H‐J . Development of resveratrol‐loaded herbal extract‐based Nanocomposites and their application to the therapy of ovarian cancer. Nanomaterials. 2018;8(6):384. 10.3390/nano8060384 PMC6027326 29857475

[162] Kalantari H , Das DK . Physiological effects of resveratrol. Biofactors. 2010;36(5):401‐406. 20623511 10.1002/biof.100

[163] Sarkar FH , Li Y , Wang Z , Kong D . Cellular signaling perturbation by natural products. Cell Signal. 2009;21(11):1541‐1547. 19298854 10.1016/j.cellsig.2009.03.009 PMC2756420

[164] Li H , Wu WKK , Zheng Z , et al. 3, 3′, 4, 5, 5′‐pentahydroxy‐trans‐stilbene, a resveratrol derivative, induces apoptosis in colorectal carcinoma cells via oxidative stress. Eur J Pharmacol. 2010;637(1‐3):55‐61. 20399769 10.1016/j.ejphar.2010.04.009

[165] Jose S , Anju SS , Cinu TA , Aleykutty NA , Thomas S , Souto EB . In vivo pharmacokinetics and biodistribution of resveratrol‐loaded solid lipid nanoparticles for brain delivery. Int J Pharm. 2014;474(1–2):6‐13. 25102112 10.1016/j.ijpharm.2014.08.003

[166] Li Y , Lokitz BS , Armes SP , McCormick CL . Synthesis of reversible shell cross‐linked micelles for controlled release of bioactive agents. Macromolecules. 2006;39(8):2726‐2728.

[167] Aryal S , Prabaharan M , Pilla S , Gong S . Biodegradable and biocompatible multi‐arm star amphiphilic block copolymer as a carrier for hydrophobic drug delivery. Int J Biol Macromol. 2009;44(4):346‐352. 19428465 10.1016/j.ijbiomac.2009.01.007

[168] Summerlin N , Soo E , Thakur S , Qu Z , Jambhrunkar S , Popat A . Resveratrol nanoformulations: challenges and opportunities. Int J Pharm. 2015;479(2):282‐290. 25572692 10.1016/j.ijpharm.2015.01.003

[169] Bawa R . Regulating nanomedicine‐can the FDA handle it? Curr Drug Del. 2011;8(3):227‐234. 10.2174/156720111795256156 21291376

[170] Nguyen AV , Martinez M , Stamos MJ , et al. Results of a phase I pilot clinical trial examining the effect of plant‐derived resveratrol and grape powder on Wnt pathway target gene expression in colonic mucosa and colon cancer. Cancer Manag Res. 2009;1:25. 21188121 PMC3004662

[171] Howells LM , Berry DP , Elliott PJ , et al. Phase I randomized, double‐blind pilot study of micronized resveratrol (SRT501) in patients with hepatic metastases—safety, pharmacokinetics, and pharmacodynamics. Cancer Prev Res. 2011;4(9):1419‐1425. 10.1158/1940-6207.CAPR-11-0148 PMC3173869 21680702

[172] Brown VA , Patel KR , Viskaduraki M , et al. Repeat dose study of the cancer chemopreventive agent resveratrol in healthy volunteers: safety, pharmacokinetics, and effect on the insulin‐like growth factor axis. Cancer Res. 2010;70(22):9003‐9011. 20935227 10.1158/0008-5472.CAN-10-2364 PMC2982884

